# Prediction limits of mobile phone activity modelling

**DOI:** 10.1098/rsos.160900

**Published:** 2017-02-15

**Authors:** Dániel Kondor, Sebastian Grauwin, Zsófia Kallus, István Gódor, Stanislav Sobolevsky, Carlo Ratti

**Affiliations:** 1 SENSEable City Laboratory, Massachusetts Institute of Technology, Cambridge, MA, USA; 2 Ericsson Research, Budapest, Hungary; 3 Department of Physics of Complex Systems, Eötvös Loránd University, Budapest, Hungary; 4 Center for Urban Science + Progress, New York University, New York, NY, USA

**Keywords:** mobile phone network, urban spatial structure, activity prediction, event detection

## Abstract

Thanks to their widespread usage, mobile devices have become one of the main sensors of human behaviour and digital traces left behind can be used as a proxy to study urban environments. Exploring the nature of the spatio-temporal patterns of mobile phone activity could thus be a crucial step towards understanding the full spectrum of human activities. Using 10 months of mobile phone records from Greater London resolved in both space and time, we investigate the regularity of human telecommunication activity on urban scales. We evaluate several options for decomposing activity timelines into typical and residual patterns, accounting for the strong periodic and seasonal components. We carry out our analysis on various spatial scales, showing that regularity increases as we look at aggregated activity in larger spatial units with more activity in them. We examine the statistical properties of the residuals and show that it can be explained by noise and specific outliers. Also, we look at sources of deviations from the general trends, which we find to be explainable based on knowledge of the city structure and places of attractions. We show examples how some of the outliers can be related to external factors such as specific social events.

## 1. Introduction

With advances in infocommunication technologies, data collection and analysis applications, it has recently became possible to study the dynamics of human activity on unprecedented scales [1–3]. With the rapid rate of urbanization, applications of this new data can be crucial in handling the challenges faced by communities in the twenty-first century. Research involving telecommunications and social network datasets augmented with spatial information can be valuable in understanding the spatio-temporal dynamics within a city. Previous work includes the analysis of mobility [4–15], the structure of social networks as represented in interactions captured by various mobile devices [8,16–20], the structure of urban environments [21–29], economic implications of these [9,30–32] and establishing the basis for a possible data-driven modelling of social and economical phenomena in cities [33–35]. A remarkable finding among these studies is that a high degree of regularity can be established in human activities [4,5,23,36], despite seemingly irregular, ‘bursty’ behaviour apparent in many cases [4,37–39].

In this paper, we focus on the regularity of human activities on urban scales as reflected by telecommunications data. In the past years, several studies have shown that it is possible to use telecommunication data to get a fresh view at the spatio-temporal dynamics within a city [21–23,27,29]. Most of these papers focused on gaining insights about the structure of a city based on the *typical* behaviour of people as extracted from their communication activities. Complementing these studies, now we focus on the question of how well such a typical pattern describes the behaviour of the city, and investigate what further insights we can gain about it by looking at the residual activities, extending upon some of the ideas presented in [15]. Using a mobile traffic dataset from London spanning 10 months, we evaluate several options for extracting a typical pattern, and compare their performances with respect to predicting the actual activity throughout our measurement period. We then focus on characterizing deviations and examining the effect of traffic volume and the spatial scale used. Focusing on large deviations, we show examples when these can be explained by external factors, such as special events in the city [40].

## 2. Data description

### 2.1. Mobile phone dataset

Our analysis is based on 3G mobile traffic data (including all kinds of devices like phones, tablets, etc.) corresponding to a statistically significant part of the total 3G mobile traffic in the Inner London area and some of the further suburbs from Greater London (most notably the western boroughs including Heathrow Airport; see figure 7 for a map of the area included), roughly several million subscribers (precise penetration rates cannot be given for confidentiality reasons). Data were collected between 1 April 2013 and 31 January 2014, and aggregated at 15 min intervals at antenna level and therefore did not reveal any individual user information. More description on the data was presented previously in our paper in [23] and is also given in electronic supplementary material, figures S17 and S18 for characterization of the general spatial distribution, correlation with census-based population estimates and the related discussion.

The 10 months of data consist of counter data recording the numbers of voice calls (Calls), text messages (SMS), unique users using an antenna at a given time (Users; this includes devices that have any activity during a 15 min interval; due to the technical constraints of how networks operate, position of inactive devices is not recorded), Requests (for data communication initiated either by the users or some applications running in the background in their mobile devices) as well as the volume of data uploaded and downloaded by subscribers (denoted by ‘UL Data’ and ‘DL Data’ hereafter). All data were scaled by an unknown factor to further protect the privacy of users and the network operator.

### 2.2. Spatial aggregation

As antenna-level data still contains noise and patterns which arise from technical features of cellular network operation, we aggregate it into various relevant spatial units. Also, we use several different levels of spatial aggregation to examine if larger spatial scales result in more regular average behaviour. We use the location of antennas as a basis for this, i.e. we associate activity from an antenna to the spatial unit it is contained in. We note that there is some uncertainty in the location of antennas in the dataset due to confidentiality reasons, while the effective size and shape of a reception area of each antenna can vary significantly according to the location and operational concerns; to this end, spatial aggregation of activities will be an approximation of real activities in the selected area; on the other hand, we believe that the distribution of volumes and observed regularities at various levels of spatial aggregation are representative in terms of regularities of urban activities on various different spatial scales. Having considered these limitations, in the following, we use five levels of spatial aggregation:

1. *Whole city*: To get an overall picture of the relevant features in the activity time series, we first aggregate all antenna-level time series and look at the Greater London area as a single spatial unit.
2. *Boroughs*: We use the official division of the Greater London area into large spatial units (median area: 39 *km*^2^) [41]. In total, we have 29 such boroughs with measurements in them out of a total of 33 boroughs making up Greater London.
3. *Wards*: We use the official division of the Greater London area into small spatial units (median area: 1.87 *km*^2^). In total, we have 381 such wards with measurements in them.
4. *Pixels*: We overlay a 500×500 *m* grid on the area of Greater London, and aggregate the data from mobile towers into the grid pixels that contains them. To reduce noise, we apply a spatial smoothing, averaging data in 3×3 blocks of pixels. In total, we have 2373 pixels with measurements in them after leaving out the bottom 5% in terms of activity volume as a further means to reduce noise.
5. *Clusters*: We use six functional clusters of London obtained by employing methods presented in [23] to group pixels together based on the similarity of their normalized activity time series. The clusters represent areas which we classify as core business, commercial, mixed (commercial/residential), residential, commuter and residential/leisure; clusters and their representative typical time series are displayed in electronic supplementary material, figure S12.

## 3. Results

### 3.1. City-wide trends

**Figure 1.**
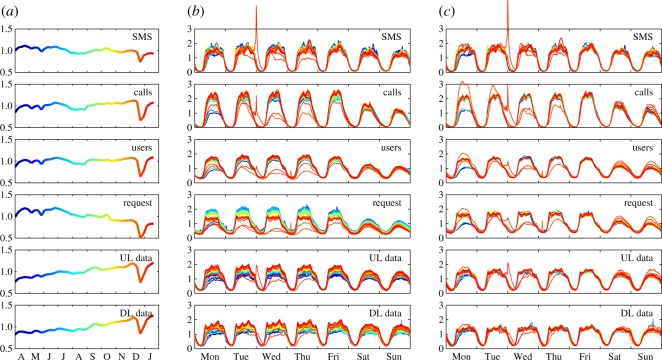
Activity trends in Greater London. (*a*) Weekly aggregated activity timelines, showing the long-term trends. (*b*) 15-min aggregated activity timelines showing the patterns for each week in the dataset. For each activity type, the timeline is normalized by its global average. Different colours correspond to the different weeks, in correspondence with colours used in (*a*). (*c*) Same as (*b*), except that for each week, the timeline is normalized by its mean over the week. Note the especially large peaks in Calls, SMS and upload volume at New Year’s Eve in (*b*) and (*c*).

In order to get a preliminary understanding of the data, we first plot the city-wide activity timelines at different temporal aggregation levels. Figure 1*a*, showing weekly aggregated timelines, emphasizes long-term trends. Most significantly, we observe a steady increase in UL and DL Data traffic accompanied by a decrease in the Request traffic; the former corresponds to the general trend of rapidly increasing mobile data usage worldwide [42], while we speculate that the latter is a consequence of changing habits in how people use phones and how network operators configure their equipment adapting to increasing demands of users. All datatypes display a slight decrease in activity in the summer, and a larger decrease around winter holidays, when people are more interested in gifts and visiting relatives than work or regular social activities, consequently being less active on the mobile networks. However, note the peaks in SMS and calls at New Year’s Eve and the following morning. Comparing weekly timelines (figure 1*b*–*c*), we see that most variation is in the volume, while the shape of a week’s time series is relatively similar during the whole period.

**Figure 2.**
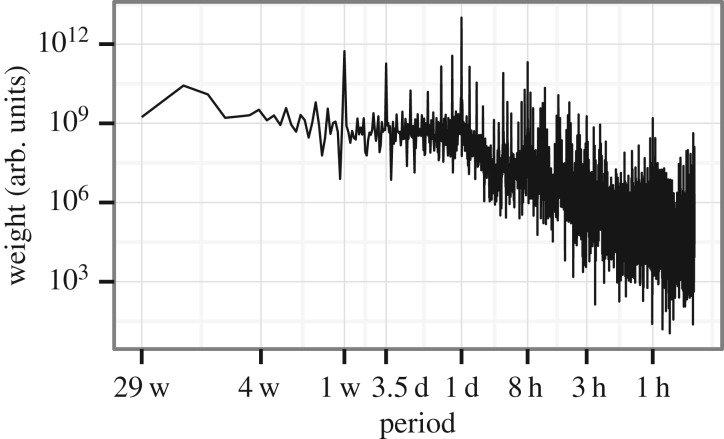
Power spectrum of city-wide activity time series for Calls. Note that the axes are on a logarithmic scale (and the *x*-axis displays the period of the component, with abbreviation of units, i.e. ‘w’ for week, ‘d’ for day and ‘h’ for hour). The period with highest weight is 1 day followed by 1 week and then several components which result in the weekday/weekend pattern. See the electronic supplementary material for other data types and a list of the most significant periods.

To complement this qualitative analysis, we look at the power spectrum of the time series to reveal the main sources of periodicity (figure 2; electronic supplementary material, figure S1 and table S1). Not surprisingly, we find that the most significant component corresponds to one day, the most important periodic aspect in the life of humans. This is followed by the period of one week and several harmonics which are required to divide the week into weekdays and weekends. Periods of 1, 8 and 12 h are also significant, which correspond to patterns describing the daily routine of people. Note that since our measurement period is less than a year, we cannot assess the relative importance of the yearly cycle; on the other hand, while the dataset spans 10 months, we did not find a monthly period to be significant.

### 3.2. Extracting typical activity time series

In this section, we present different ways to compute the typical activity patterns. For each activity type *λ* (SMS, Calls, Users, Request, UL Data or DL Data), let $A_{i}^{\lambda }(w,n,t)$ be the vector representing the total amount of activity of each 15 min interval *t* of each day *n* of week *w* in the dataset in spatial unit *i* (*t*∈{1,2…96}, *n*∈{1,2…7}, *w*∈{1,2…45}). In accordance with the different spatial aggregation levels presented previously, the *i* index can represent any spatial unit on any of the aggregation levels presented there. At first, we look at the timeline aggregated over the whole city, while later on, we will turn to activity time series in all the different spatial aggregation units. In this paper, we evaluate the following choices for typical activity patterns so as to separate them from residual activity and predict activity levels in our data:

#### 3.2.1. Typical day

The basic temporal unit in our lives is a day; based on this, we expect the daily cycle to have a strong effect on the mobile network activity patterns. Indeed, if we look at the Fourier-spectrum (figure 2; electronic supplementary material, figure S1), we see that the daily period is present with the largest amplitude. Based on this, the simplest way to model the activity time series is to use the average of measured activities in the same 15 min interval of each day in our dataset as an estimation for activity levels at a specific time:

3.1 $$M_{i}^{\lambda }(w,n,t)={\left\langle A_{i}^{\lambda }(w^{\prime },n^{\prime },t)\right\rangle }_{\left(w^{\prime },\;n^{\prime })\in \{\mathrm{all}\;\mathrm{days}\right\}}.$$

#### 3.2.2. Typical weekday and weekend

Apart from a daily period, human behaviour shows a strong weekly pattern; the most important aspect of this is that there is an obvious difference between weekdays and weekends. We can account for this with modelling the activity time series on weekdays and weekends independently:

3.2 $$M_{i}^{\lambda }(w,\mathrm{weekday},t)=\langle A_{i}^{\lambda }(w^{\prime },n^{\prime },t)\rangle {|}_{\begin{array}{c} w^{\prime }\in \{\mathrm{all}\;\mathrm{weeks}\}\\ n^{\prime }\in \{\mathrm{weekdays}\} \end{array}}$$

and

3.3 $$M_{i}^{\lambda }(w,\mathrm{weekend},t)=\langle A_{i}^{\lambda }(w^{\prime },n^{\prime },t)\rangle {|}_{\begin{array}{c} w^{\prime }\in \{\mathrm{all}\;\mathrm{weeks}\}\\ n^{\prime }\in \{\mathrm{weekends}\} \end{array}}.$$

#### 3.2.3. Typical week

Apart from the difference between weekdays and weekends, there are smaller but significant differences among weekdays and between Saturday and Sunday. We can thus improve the model of typical activity patterns by using the week as a basic unit, and modelling activity time series on different days separately, as an average of activities only on that day:

3.4 $$M_{i}^{\lambda }(w,n,t)={\left\langle A_{i}^{\lambda }(w^{\prime },n,t)\right\rangle }_{w^{\prime }\in \{\mathrm{all}\;\mathrm{weeks}\}}.$$

We note that the choice of one week as a basic unit for aggregation and statistical analysis was intuitively used in several studies previously [15,40].

#### 3.2.4. Estimates with trends

So far we have only included typical patterns, i.e. we modelled human activity as a perfectly periodic pattern. Looking at figure 1, however, it is obvious that there are long-term trends which are missed by this approach. A simple way to account for this is to introduce a daily or weekly scaling factor in the previous models. For example, in the case of of the typical week (equation (3.4)), this can be achieved as the following form:

3.5 $$M_{i}^{\lambda }(w,n,t)=\Gamma_{i}(w){\left\langle A_{i}^{\lambda }(w^{\prime },n,t)\right\rangle }_{w^{\prime }\in \{\mathrm{all}\;\mathrm{weeks}\}.}$$

Here *Γ*_*i*_(*w*) is a weekly scaling factor which can be chosen according to many possibilities (note that in the case of the typical day and typical weekday and weekend models this factor will also depend on the day, i.e. we would include a *Γ*_*i*_(*w*,*n*) term in equations (3.1) or (3.3)). Possible choices could include a fitted function describing the trends (i.e. if we consider regular/yearly trends like the effect of holidays or people going on vacations which do not change over different years, e.g. for calls), an extrapolation of expected future trends (e.g. for data traffic volume which is dynamically increasing), or the result of a model predicting the possible future of the time series based on the past. The main point is separating the original problem of modelling the time series into two independent problems: modelling the shape of the weekly time series and modelling the long-range trends. The simplest choice is using the observed weekly amplitudes through our measurement period:

3.6 $$\Gamma_{i}(w)=\frac{\sum_{n=1}^{7}\sum_{t=1}^{96}A_{i}^{\lambda }(w,n,t)}{\sum_{n=1}^{7}\sum_{t=1}^{96}{\left\langle A_{i}^{\lambda }(w^{\prime },n,t)\right\rangle }_{w^{\prime }\in \{\mathrm{all}\;\mathrm{weeks}\}}}.$$

With such a choice of weekly scaling factor, we essentially limit our focus to estimating the shape of the weekly time series. However, we feel that modelling the long-term trends in mobile network traffic volume is beyond the scope of the current work due to the limited measurement period.

#### 3.2.5. Sliding window average model

A possible variation of the previous model is that instead of using amplitudes for each week, we model typical activity with the average calculated using a shorter symmetric time window centred around the date of interest. This way we hope to account for possible trends in both the shape and amplitude of the time series, instead of only for the amplitude as in the previous case:

3.7 $$M_{i}^{\lambda (k)}(w,n,t)={\left\langle A_{i}^{\lambda }(w,n,t)\right\rangle }_{w^{\prime }\in \{w-k,w-k+1\ldots w+k\}}.$$

Here $k\in N^{+}$, and *W*:=2*k*+1 is the size of the window as measured in weeks. A notable disadvantage of this method is that only the central time interval can be modelled, a total of 2*k* weeks is lost at the beginning and the end of the data collection period. Nevertheless, as our measurements cover a relatively long period of time, we can test several different values of *W*. In our analysis, we studied examples in the range of *W*∈{3,5,…,17}. To have consistent results, we consider results from a common central time interval (i.e. we leave out 9+9 weeks).

**Figure 3.**
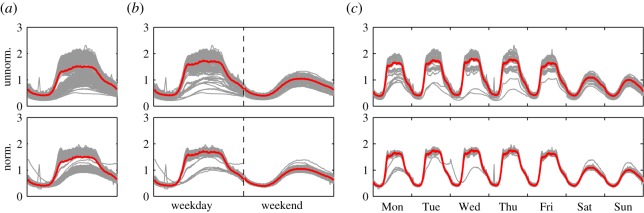
Comparing the different aggregation methods on the Request data. Grey curves are individual daily or weekly activity patterns, while the red curve is the typical activity obtained as an average of the individual patterns. (*a*) Typical day, (*b*) typical weekday and weekend and (*c*) typical week. Top row: original data; bottom row: data normalized by the daily and weekly average activity, showing that there is much less variation in the shape of the time series than in the volume, suggesting that a model where trends are included with either a daily or weekly scaling factor can result in significantly improved performance.

The process of aggregation of data is illustrated in figure 3.

### 3.3. Estimating the regularity of typical patterns

To quantify how typical activity patterns relate to the actual patterns, in the following we focus on the residuals, which at a given place and time are calculated as:

3.8 $$\delta_{i}^{\lambda }(t)\equiv A_{i}^{\lambda }(t)-M_{i}^{\lambda }(t).$$

Since *δ* will depend on the absolute activity which varies greatly in time and space, we define the *predictability score* of a location and time, which is thus a measure that facilitates the comparison of different locations and times:

3.9 $$\Phi_{i}^{\lambda }(t)\equiv 1-\varepsilon_{i}^{\lambda }(t),$$

where $\varepsilon_{i}^{\lambda }(t)$ is the normalized error, defined as $\varepsilon_{i}^{\lambda }(t)\equiv 2(|A_{i}^{\lambda }(t)-M_{i}^{\lambda }(t)|/(A_{i}^{\lambda }(t)+M_{i}^{\lambda }(t)))$. Note that due to their definitions, these will be bounded, i.e. *ε*∈[0,2] and *Φ*∈[−1,1]. We note that we chose the predictability scores as a measure of evaluation as they give a convenient measure of how much of the activity is accounted for by the typical activity patterns defined in our models. While the purpose of the models presented in the last section is to characterize the regularity of human behaviour as represented in mobile network usage patterns and not the actual prediction of future activity (this is represented in the conceptual choice of using data from the whole time period as one unit to construct the typical activity patterns instead of evaluating metrics for future activities based on a model incorporating past activities as parameters), the predictability scores presented here also serve as guidelines and possible upper bounds for the predictive power of models based on typical patterns and seasonal variation. We expect that these could possibly be superseded by a prediction framework based on nonlinear time series analysis. For comparison, in the electronic supplementary material, we also present results for characterizing regularities with a more traditional measure of normalized errors defined as $|\delta_{i}^{\lambda }(t)|/M_{i}^{\lambda }(t)$ (electronic supplementary material, figures S4, S11 and S15 and tables S2, S4, S6 and S8).

Apart from looking at residuals or individual predictability scores in different locations and at specific times, we can get an overall view on the models’ behaviour by calculating averaged predictability scores at various possible spatial and temporal resolutions. To decrease the effect of noise present in low activity intervals or places, we take weighted averages, weighting the predictability score at a given time and place with the total activity there:

3.10 $$\langle \Phi \rangle =\frac{{\left\langle A_{i}(t)\Phi_{i}(t)\right\rangle }_{i,t}}{{\left\langle A_{i}(t)\right\rangle }_{i,t}}.$$

Of course, we can choose to limit the averages to only a subset of times and places, e.g. calculate the average predictability at a given place or only on a given day.

**Figure 4.**
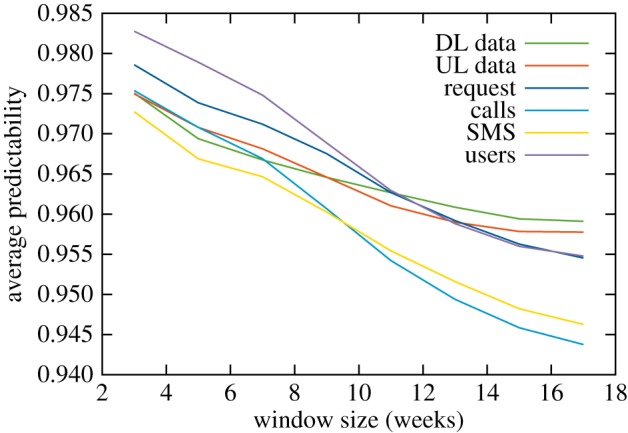
Predictability scores using the sliding window average model, average predictability as a function of the window size used.

To get a general picture of variability explained by typical activities, we first calculate average predictability scores arising when applying the estimates to the aggregated timeline of mobile network activity in the Greater London area. These results are summarized in table 1. What we see is that all cases result in relatively high predictability scores corresponding to the general high regularity of human activities; also, significant improvements can be gained from including more detail, e.g. taking into account the differences in days of the weeks and long-term trends. Also, the especially high predictability values gained with the sliding window model highlight that on the level of the whole city, slow and steady changes have higher effect than individual irregularities (see also figure 4). In the following, we concentrate on estimating activities with the typical week including trends (i.e. equations (3.5) and (3.6)); also, based on the observation of holidays having a high effect on predictability results, we omit the last two months of data, which are highly affected by the Christmas season (figure 1); accounting for these effects would require data from a longer data collection period to be able to model yearly periodicity and long-term trends. On the other hand, our aim here is mainly to look at the regularity of everyday activities.

**Table 1.** Predictability scores for the different tested models at the city-wide scale (i.e. 〈*Φ*〉, values closer to one means more regular activity time series). The values in parenthesis correspond to the case when we do not take the civic holidays into account in the analysis.

	typ. day		typ. weekday & weekend		typ. week		sliding wind. typ. week	
type	original	with trends	original	with trends	original	with trends	3-week	17-week
SMS	0.815 (0.825)	0.822 (0.830)	0.895 (0.913)	0.907 (0.921)	0.905 (0.926)	0.918 (0.936)	0.976 (0.977)	0.960 (0.963)
Calls	0.677 (0.689)	0.691 (0.700)	0.866 (0.888)	0.891 (0.906)	0.897 (0.918)	0.925 (0.941)	0.976 (0.978)	0.958 (0.962)
Users	0.831 (0.848)	0.841 (0.852)	0.927 (0.951)	0.944 (0.958)	0.936 (0.963)	0.953 (0.971)	0.977 (0.98)	0.952 (0.959)
Request	0.764 (0.828)	0.775 (0.831)	0.850 (0.939)	0.868 (0.947)	0.854 (0.952)	0.873 (0.960)	0.978 (0.98)	0.947 (0.956)
UL data	0.841 (0.868)	0.848 (0.871)	0.896 (0.942)	0.907 (0.948)	0.899 (0.948)	0.910 (0.955)	0.972 (0.974)	0.948 (0.951)
DL data	0.856 (0.897)	0.861 (0.900)	0.888 (0.944)	0.895 (0.949)	0.891 (0.953)	0.898 (0.958)	0.984 (0.987)	0.957 (0.964)

**Figure 5.**
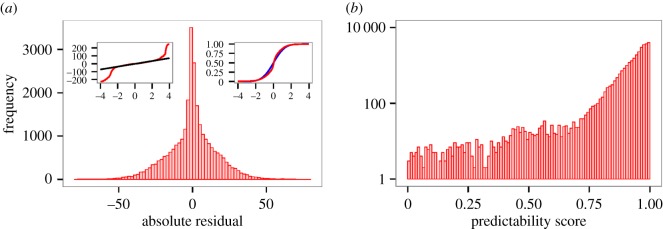
Distribution residuals for city-wide Calls data. Absolute residuals (*a*) and predictability scores (*b*). The insets in (*a*) show a test for normal distribution. Especially, the quantile plot in the left inset shows that there are a significant number of outliers suggesting systematic differences from the notion of uncorrelated noise. For more discussion, see electronic supplementary material, figure S2.

To get a more detailed view on the ability to model human activities in the city, at first, still on the level of the whole city time series, we look at the distribution of individual absolute residuals (i.e. errors at specific times, given by the *δ*_*i*_(*t*) values defined in equation (3.8)). For all data types, we have fast decreasing distributions, however, with a significant number of outliers. For all data types, small errors approximately follow a normal distribution, while larger errors can be thought of as a significant number of outliers in this picture (figure 5 for the distribution in the case of Calls volume and electronic supplementary material, figure S2 for all datatypes and more discussion). We speculate that there are indeed two factors behind this: deviation from the typical timeline is the result of general noise (which is expected to follow a normal distribution with parameters possibly varying in time and space) and also more specific factors such as special events or the weather. On the other hand, the distribution of the predictability scores is better approximated by an exponential distribution (figure 5 for Calls; electronic supplementary material, figure S3 (for all data types) and figure S4 (for the distribution of relative errors)).

**Figure 6.**
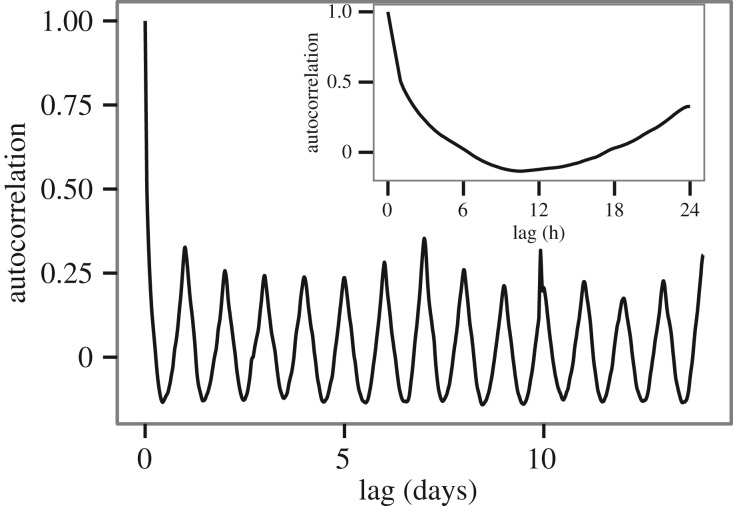
Temporal autocorrelation of normalized errors. Autocorrelation between the errors at given time intervals apart. The inset shows the same data on the scale of 24 h, i.e. correlation between errors separated by a given amount of time. The autocorrelation function shows a rapid decrease to zero, followed by a periodic pattern corresponding to the general daily periodicity in data (see the electronic supplementary material for more explanation). The inset shows the same data on the scale of 1 day, showing that autocorrelation reaches zero in less than 6 h, which can be interpreted as the typical time scale of a special event which disrupts the regular habits of people.

Next, we examine if there is significant temporal autocorrelation among normalized residuals calculated at different times. We find that generally, temporal correlation is only present on short time scales, i.e. a few hours. This means that the employed typical week model indeed captures most of the deterministic part of activity time series; apart from that, disturbances from the typical activity span a few hours in time, but variations in predictability on longer time scales can be considered independent. These results are illustrated in figure 6 for predictability scores in the volume of Calls and in electronic supplementary material, figures S5 and S6 for all other activity types, which also yield very similar results. The oscillating behaviour on longer time scales can be explained by an apparent positive correlation between traffic volume and predictability scores (i.e. relative residuals decrease with increasing volume, as expected based on the central limit theorem). In electronic supplementary material, figures S8, S9 and S10, predictability scores are presented as a function of time of the week and also as a function of activity volume, while in electronic supplementary material, figure S11, relative errors are displayed as a function of activity volume; there is a clear weekly cycle which closely resembles the cycle seen in activity volume, and also a positive correlation between volume and predictability scores can be established, while relative errors correlate negatively with volume.

### 3.4. Spatial limits of predictability

Having evaluated estimates on regularity on the city-level time series, we now turn the time series from lower levels of spatial aggregation. Using the typical week with trends estimation (equations (3.5) and (3.6)) and the eight-month period between April and November 2013, we ask the question if local activity time series show a similar degree of regularity as the aggregated time series on the whole city level.

We calculate the typical week model in every spatial unit at each spatial aggregation level and use it to calculate average predictability scores. We calculate average predictability scores using two approaches. In the first case, we use the typical week model at each aggregation level to predict the time series at the same aggregation level. This way we test if activity time series get harder to predict if we are interested in it at specific locations. In the second case, we use the typical week model computed at various spatial aggregation levels to predict the time series of pixels inside the specific larger spatial units. This way, we test for the significance of individual variations on smaller scales.

We display the results for various spatial resolutions in table 2, showing average predictability scores according to equation (3.10). In panel (*a*), we show results for predicting time series in the same spatial units where the model was calculated. What we clearly see is that predictability increases as we increase the area included, meaning that irregularities tend to ‘cancel out’ if we aggregate larger areas together. In panel (*b*), we show results for predicting the pixels’ time series using the typical week model calculated for larger spatial units containing the given pixel. As expected, larger areas give worse results, as more pixels with possibly different timelines are averaged. An exception is the case of the clusters, which give better results than the boroughs (and for some data types, even better results than the wards) despite being bigger than them, as these were explicitly constructed to contain similar pixels. We give further estimates in electronic supplementary material, table S2, where averages without weights, standard deviations and average relative deviations are also displayed, providing results consistent with table 2 and the above discussion.

**Table 2.** Average predictability scores at various spatial aggregation levels. (*a*) Predictability increases as we look at bigger spatial units, as the relative variance of the aggregated data decreases. (*b*) Possibility to predict the time series at the lowest spatial resolution level (i.e. the 500×500 *m* grid pixels) using the models calculated at bigger spatial levels containing the pixel.

	(*a*) predictability at various spatial resolution					(*b*) predictability of pixels using higher resolution data				
type	pixels	wards	boroughs	clusters	city	pixels	wards	boroughs	clusters	city
DL data	0.811	0.794	0.925	0.953	0.966	0.811	0.789	0.775	0.791	0.754
UL data	0.76	0.740	0.900	0.946	0.968	0.76	0.738	0.727	0.739	0.707
Request	0.898	0.891	0.942	0.951	0.971	0.898	0.842	0.808	0.851	0.738
Calls	0.91	0.904	0.953	0.956	0.968	0.91	0.887	0.872	0.884	0.85
SMS	0.824	0.812	0.914	0.938	0.958	0.824	0.801	0.788	0.802	0.763
Users	0.927	0.922	0.961	0.963	0.980	0.927	0.88	0.843	0.888	0.787

**Figure 7.**
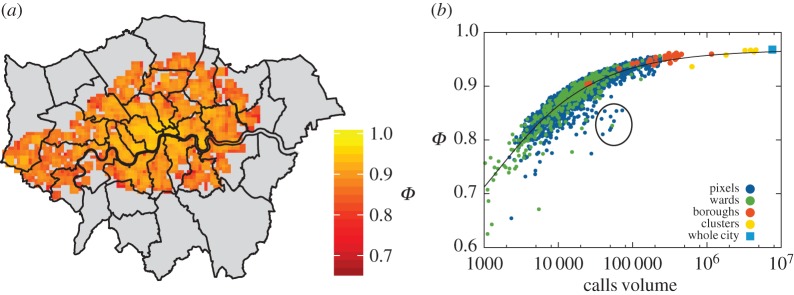
Spatial distribution of average predictability scores. (*a*) Average predictability scores over London (in the 500×500 *m* grid pixels). (*b*) Predictability scores at various aggregation levels as a function of the total activity volume. We see a clear trend with higher levels of activity resulting in higher average predictability scores; in the case of the pixels, the two measures have a Pearson’s correlation coefficient of 0.6121963 and a Spearman’s rank-correlation coefficient of 0.9032438, and can be fitted with the functional form $\Phi =0.968-10.8/\sqrt{A+798}$. Note that the fit was performed with taking only the pixels into account, but it also approximates the trend for the other spatial aggregation levels. The group of ‘outlier’ pixels (circled) can be identified as the vicinity of the Wembley Stadium, a major sports and event venue, where we can expect high volume of activity with less regularity. Map was generated with R, using the ggplot2, sp and rgdal libraries [43]; the background map was generated from the digital maps (shapefiles) available from the Greater London Authority [41]. Additional processing for the legend was done using Inkscape [44].

Apart from calculating city-wide averages, we also look at the distribution of predictability values on smaller scales. We display the overall spatial distribution of predictability of Calls volume in the city at the pixel level in figure 7. Not very surprisingly, we find that average predictability is slightly higher in the city centre, and there are some pixels with especially low predictability in the suburbs. Using the functional clusters to interpret the spatial distribution, we can conclude that predictability is generally higher in business and commercial areas, slightly less in residential areas and even less in areas with relative high activity from commuters and in the cluster identified as leisure areas, which contains the most significant sports venues in the Greater London area (see electronic supplementary material, figure S12 for a map of London displaying the locations of the clusters and the associated typical activity timelines; see electronic supplementary material, tables S3–S8 for cluster-wide average predictability scores and relative deviations). Based on the general expectation that the relative amount of noise should decrease when more activity is aggregated and our previous observations of this and that of higher activity volumes also resulting in higher predictability scores (see electronic supplementary material, figures S8–S11 for city-wide evaluation), we expect that some of these variations can be explained by variations in activity volumes. To test this, in figure 7*b*, we display the average predictability scores for calls as the function of the total volume in all spatial aggregation units. There is a clear correlation, with data from all spatial aggregation levels following the same trend. Based on the central limit theorem, for purely uncorrelated noise, we would expect the functional form $\Phi ∼1-\sqrt{A/A_{0}}$ with an appropriate *A*_0_ normalization constant. As discussed previously, residuals display significant deviations from a normal distribution, especially in that there are many outliers (see also electronic supplementary material, figure S2); taking the approximation that the volume does not affect the relative magnitude of these, we approximate the trend in average predictability due to white noise with the following form: $\Phi =1-\Delta -a_{0}/\sqrt{A+a_{1}}$. The best fit for the predictability values of the pixels is displayed in figure 7; it also describes the trend at different spatial aggregation levels remarkably well, which was not the case of other possible power-law fits of the data. In this case, the *Δ* parameter can be interpreted as a limit on regularity or predictability due to specific variations (in contrast with uncorrelated noise). Constraining *Δ*≡0 gave much worse results in all cases considered, meaning that an assumption of *Δ*>0 is plausible in this case; the fitted value for calls volume is *Δ*=0.029. Considering the different data types, the general trend is very similar in all cases with varying parameters, except for data requests and unique active user numbers, where a power-law fit in the form of *Φ*=1−*a*_0_/*A*^*β*^ also seems equally plausible, with the values of the *β* exponent being 0.17 and 0.21, respectively (see electronic supplementary material, figures S13 and S14 for details, and electronic supplementary material, figure S15 for the same plot for relative errors).

### 3.5. Detecting special events and locations

Having established the dependence of regularity and predictability on the average volume, we now look at deviations from the trend in more detail. So far, we have looked at regularity at various spatial aggregation levels and found that it depends on the level of aggregation: irregularities cancel out on larger spatial scales. While lower averaged predictability scores can be explained with the activity patterns being more irregular at smaller scales, or lower activity levels, it can also be the effect of a social event, a large-scale disruption or generally any significant change in the daily routine of mobile phone users. Identifying these can be useful for many different purposes for the mobile network operators, authorities, local businesses, etc. Thus our goal is to evaluate if we can efficiently detect outliers in our dataset, and interpret them in terms of events that took place in London over the course of our analysis.

**Figure 8.**
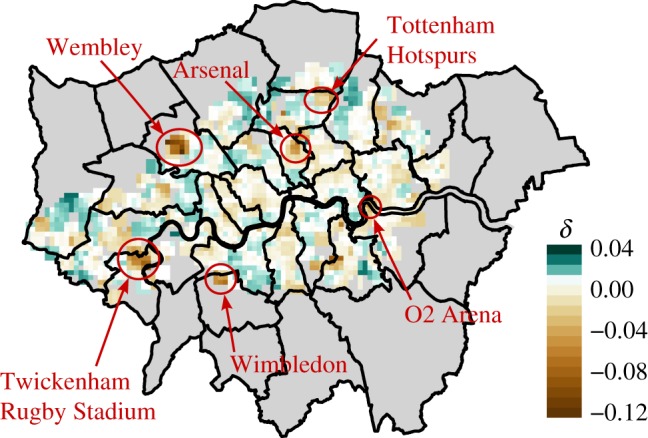
Differences of pixel level average predictability scores from the expectation based on total activity volume. Some of the negative differences (i.e. areas with unexpectedly low predictability) can be explained by the presence of large sports or concert venues located there. Map was generated with R, using the ggplot2, sp and rgdal libraries [43]; the background map was generated from the digital maps (shapefiles) available from the Greater London Authority [41]. Additional processing for the legend and markers was done using Inkscape [44].

At first, we look at the average predictability score of a pixel for the entire duration of the analysis. There are some locations where in spite of a high overall activity level the predictions still fall below the expected range. For example, in figure 7, we see a group of pixels separated. This is easily explained by the fact that these pixels contain and neighbour the Wembley Stadium, a major sports and event venue where reoccurring social events, sport and music spectacles attract significant crowds that drive up the communication activities of the area at these times, resulting in less regular patterns in general. Doing a similar analysis more systematically, in figure 8, we show the pixel level deviations from the fitted trend for the calls volume. Besides Wembley, we were able to identify further areas where predictability is significantly lower than expected as neighbourhoods containing other major sports and entertainment venues of London, where we indeed expect that the schedule of events is more irregular than the general activity pattern in other neighbourhoods.

In the following, we evaluate the possibility of temporally localized outlier analysis. For this purpose, we consider the activity in one pixel over the course of a day as a basic unit. This means that we aim to identify date and pixel pairs as outliers, i.e. cases when we can consider a specific pixel on a specific day as an outlier. To achieve this, we compute the daily average predictability score on the pixel level, and look for days and pixels where predictability scores are significantly lower than typical as possible candidates for special events.

**Figure 9.**
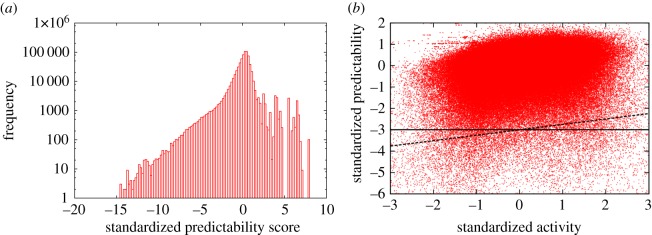
Characterizing daily predictability scores in individual pixels. (*a*) Distribution of daily average standardized predictability scores for calls on the pixel level. We see a heterogeneous distribution with an approximately double-exponential behaviour similarly to the case of the distribution of city-wide predictability scores (electronic supplementary material, figure S3). (*b*) Standardized predictability scores as the function of standardized activity (each point represents a day in a pixel; both predictability scores and activities are standardized by pixel, i.e. using the average and standard deviation from that specific pixel). We see a definite correlation; the Pearson’s correlation coefficient is 0.209982 and the Spearman’s rank-correlation coefficient is 0.275228. The black lines show the thresholds applied when identifying outliers. The horizontal line corresponds to the *z*_*i*_(*d*)<−3 threshold in the standardized predictability values. The dashed line corresponds to the adaptive threshold *z*_*i*_(*d*)<0.253*v*_*i*_(*d*)−3.

If we look at figure 7, it is clear that a city-wide predictability threshold will not be sufficient to identify outlier days, as some pixels would be classified as outliers on every day, while others never. To overcome this problem, we establish a different threshold in each pixel, and use that to identify a list of outlier days separately for that pixel. We achieve this by calculating the average and standard deviation of predictability scores on the pixel level, and use a standardization in the following: *z*_*i*_(*d*)=(*Φ*_*i*_(*d*)−〈*Φ*_*i*_〉)/*σ*_*Φ*_*i*__, where *Φ*_*i*_(*d*) is the average predictability score for pixel *i* on day *d*, and *z*_*i*_(*d*) is the obtained standardized predictability score. We then classify a day in a specific pixel an outlier if the corresponding *z*_*i*_(*d*) is below a given threshold. The aggregate distribution of *z*-scores is shown in figure 9; it displays a double exponential tail for negative *z* values, with the crossover between the two around *z*≈−2.5.

To account for the overall effect of activity volumes on predictability, we use a threshold which also depends on daily activity volume. As activity varies highly with the place, we use standardization here too: *v*_*i*_(*d*)=(*A*_*i*_(*w*,*n*)−〈*A*_*i*_(*w*′,*n*)〉_*w*′_)/*σ*_*A*_*i*__, where *A*_*i*_(*w*,*n*) is the sum of activity in pixel *i* on day *n* of week *w* (i.e. averages and standard deviations used for standardization were calculated separately for each day of the week here, as the total activity also varies significantly over the course of a week). In figure 9, we plot the standardized predictability scores *z*_*i*_(*d*) as a function of the standardized activities *v*_*i*_(*d*). We see that the two are somewhat correlated (the Pearson’s correlation coefficients is 0.20982, which we interpret as significant given the large deviation in standardized predictability scores), i.e. smaller average predictability scores are partly caused by the daily activity being less than the average. To account for this effect, we use a threshold for detecting outliers which depends on the standardized daily activity too: we consider a day in a pixel an outlier if *z*_*i*_(*d*)<0.253*v*_*i*_(*d*)−3. The slope here was determined by fitting a linear function to all of the points, while the additional −3 was determined such that for *v*_*i*_(*d*)=0 (average activity), we get back a 3*σ* threshold.

**Figure 10.**
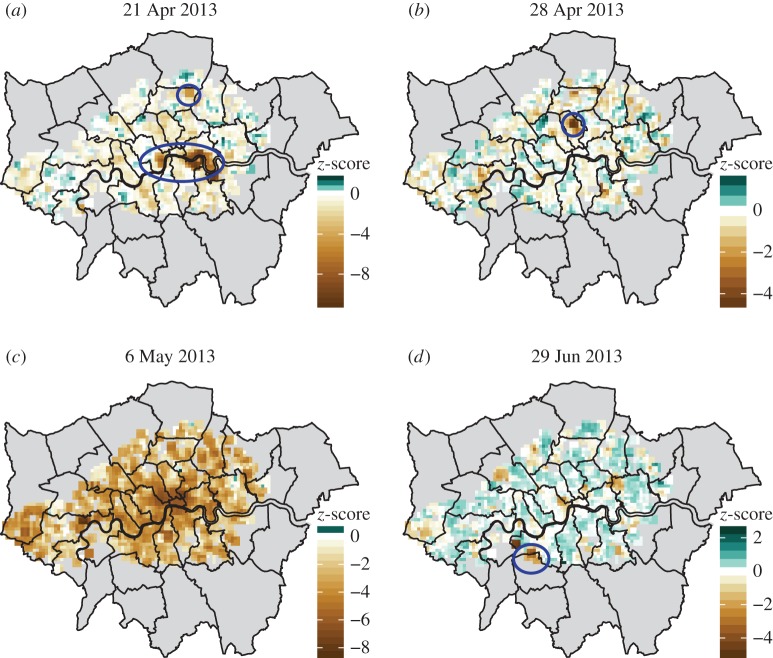
Four example days with notable outlier pixels. (*a*) 21 April, when the annual Marathon race took place in the city (middle area), while also a football match took place between the Tottenham Hotspurs and Manchester City in north London (top area). (*b*) 28 April, where the outlier pixels contain the Emirates Stadium which was the venue for a football match between Arsenal and Manchester United. (*c*) 6 May, a public holiday; in this case, predictability is low in all of the pixels and many were classified as outliers, corresponding to the fact that the general routine of people differed significantly from a normal weekday. (*d*) 29 June, the date of several significant matches in the Wimbledon tennis cup. Note that the neighbourhood containing the Wembley Stadium can be identified in this figure too as having lower than expected predictability, even though it was not below the threshold for outlier identification; indeed this was the date of a concert there which drew a significant crowd. Map was generated with R, using the ggplot2, sp and rgdal libraries [43]; the background map was generated from the digital maps (shapefiles) available from the Greater London Authority [41]. Additional processing for the legend and markers was done using Inkscape [44].

Days with especially high number of outlier pixels can be identified as global events, e.g. public holidays (1 April, 6 and 27 May, 26 August in 2013). Localized outliers whose source we could identify include important sporting events such as matches in the Wimbledon tennis cup, football matches with high attendance (e.g. the UEFA Champions League Final at Wembley on 25 May, Arsenal versus Norwich City on 13 April, Arsenal versus Manchester United on 28 April, the FA Cup Final at Wembley on 11 May or Tottenham versus Manchester City on 12 April) and large-scale social events such as the London Marathon on the 21 April. Some example events are displayed in figure 10, while the London Marathon is displayed in more detail in electronic supplementary material, figure S16. A notable feature of this event is the changing location during the day. Looking at the distribution of the predictability scores, the course of the race unfolds as the crowd of spectators closely followed the runners.

## 4. Conclusion

In this paper, we have demonstrated the possibility to decompose localized timelines of telecommunications activity into regular and residual patterns on 10 months of aggregated mobile network usage data from the Greater London area. We evaluated estimation of typical activities based on the combination of typical timelines and long-term trends, and on various spatial aggregation levels. In line with previous studies showing a high degree of regularity in human activities [4,5,23,36], we found that if we consider the whole city aggregated timelines, residuals are only on the scale of 2–4%, while they get much larger if we consider smaller neighbourhoods. We also showed that applying an activity-based clustering [23] to the smaller neighbourhoods results in much better approximation of the time series in smaller aggregation units inside the clusters. We showed that the magnitude of residuals greatly varies with volume of activity in line with expectations based on the central limit theorem, while maximum regularity for regions with high volume of activity is limited by external causes of deviations not accounted for in our simple model of regular patterns and uncorrelated noise; an analysis of the magnitude of relative deviations on different spatial aggregation levels resulted in *Δ*=2.9% as a limit for noise not explained by regular patterns or uncorrelated noise (see figure 7*b* and the related discussion). We demonstrated that the main sources of periodicity are the daily and weekly cycles, and that temporal correlations in the residual activity span only a few hours, a typical length for a special event or disruption in the daily routine of people. We were able to interpret times and places significantly deviating from the typical activity in terms of relevant local events. We expect our study to be a first step in better integrating mobile network data in urban studies both in terms of planning and in near real-time monitoring and anomaly detection which could allow early response to some abnormal situation and the cost-effective detection of relevant changes in urban activity patterns. Apart from mobile data, we expect our methodology to be easily applicable to several other data sources, allowing the exploitation of rapid advances in urban sensing technologies resulting in low-cost data available in increasing volumes.

## Supplementary Material

Supplementary Material including additional figures and tables which provide more detailed results and further description of data collection and processing

## Data accessibility

Restrictions apply due to confidentiality reasons from the data provider. A subset of the original data, including city-level mobile communication time series is available at the Dryad Digital Repository: http://dx.doi.org/10.5061/dryad.2t3t7 [45]. Raw measurements representing activities on a cellular tower basis are confidential, and property of the network operators supporting the research. Data were shared with Ericsson and the researchers involved in the project under strict non-disclosure agreements. The main reasons for operators not sharing more data are protecting network configuration details and the privacy concerns of high granularity information on local habitants. Spatially aggregated data were used as a basis of present study for the above reasons. From the various granularity levels, pixel- and ward-based data still reveals network configuration details. But at the city, borough and functional cluster levels, the datasets received authorization for public access. Hence the basic time averaging and spatial smoothing were applied as described in the article in order to create the mid-level datasets that we used and we share as discussed previously. These allow recreation of the results presented in the figures. We note that some of the data were already made available previously on the http://manycities.org website.
