# Supplementary material for: Prediction limits of mobile phone activity modelling
Source: R Soc Open Sci. 2017 Feb 15;4(2):160900. doi: 10.1098/rsos.160900 (PMC5367302; doi:10.1098/rsos.160900)
Supplement: Supplementary Material including additional figures and tables which provide more detailed results and further description of data collection and processing [file rsos160900supp1.pdf]

Prediction limits of mobile phone activity modeling

Supplementary material

Dániel Kondor[1,2,3,*], Sebastian Grauwin[1], Zsófia Kallus[2,3], István Gódor[2], Stanislav Sobolevsky[1,4], Carlo Ratti[1]

1 SENSEable City Laboratory, Massachusetts Institute of Technology, Cambridge, MA, USA
2 Ericsson Research, Budapest, Hungary
3 Department of Physics of Complex Systems, Eötvös Loránd University, Budapest, Hungary
4 Center for Urban Science + Progress, New York University, New York, NY, USA
∗ E-mail: dkondor@mit.edu

Calls		SMS		Users	
Period (hrs.)	Rel. weight	Period (hrs.)	Rel. weight	Period (hrs.)	Rel. weight
24	101	24	870	24	469
168	5.42	12	80	168	49.9
28	3.63	168	27	28	21.8
8	2.06	28	12	12	16.6
84	1.83	84	7.7	21	10.9
33	1.43	6	6.3	84	9.53
21	1.38	4.48min	6.3	33.36min	6.86
12	0.8	4872	4.8	8	3.34
18.40min	0.35	33.36min	4.7	18.40min	2.11
2436	0.27	21	3.7	4.48min	2.09

DL data		UL data		Request	
Period (hrs.)	Rel. weight	Period (hrs.)	Rel. weight	Period (hrs.)	Rel. weight
24	318	24	185	24	140
12	64.7	12	17.8	168	20.3
168	9.22	168	8.73	28	9.9
8	8.08	4872	3.32	21	4.36
4.48min	5.8	28	3.03	84	3.73
4872	4	2436	1.88	33.36min	2.84
2436	3.25	4.48min	1.85	12	2.47
12.55min	2.6	21	1.84	4872	2.33
11.12min	2.41	8	1.78	8	2.3
28	1.71	84	1.72	4.48min	0.75

Table S1: Most significant periods in the activity timelines. For all data types, the one day period has by far the largest weight. The one week period (168 hours) is either second or third (in these cases, a half-day period is second); these are followed by the components needed to obtain the weekday / weekend separation.

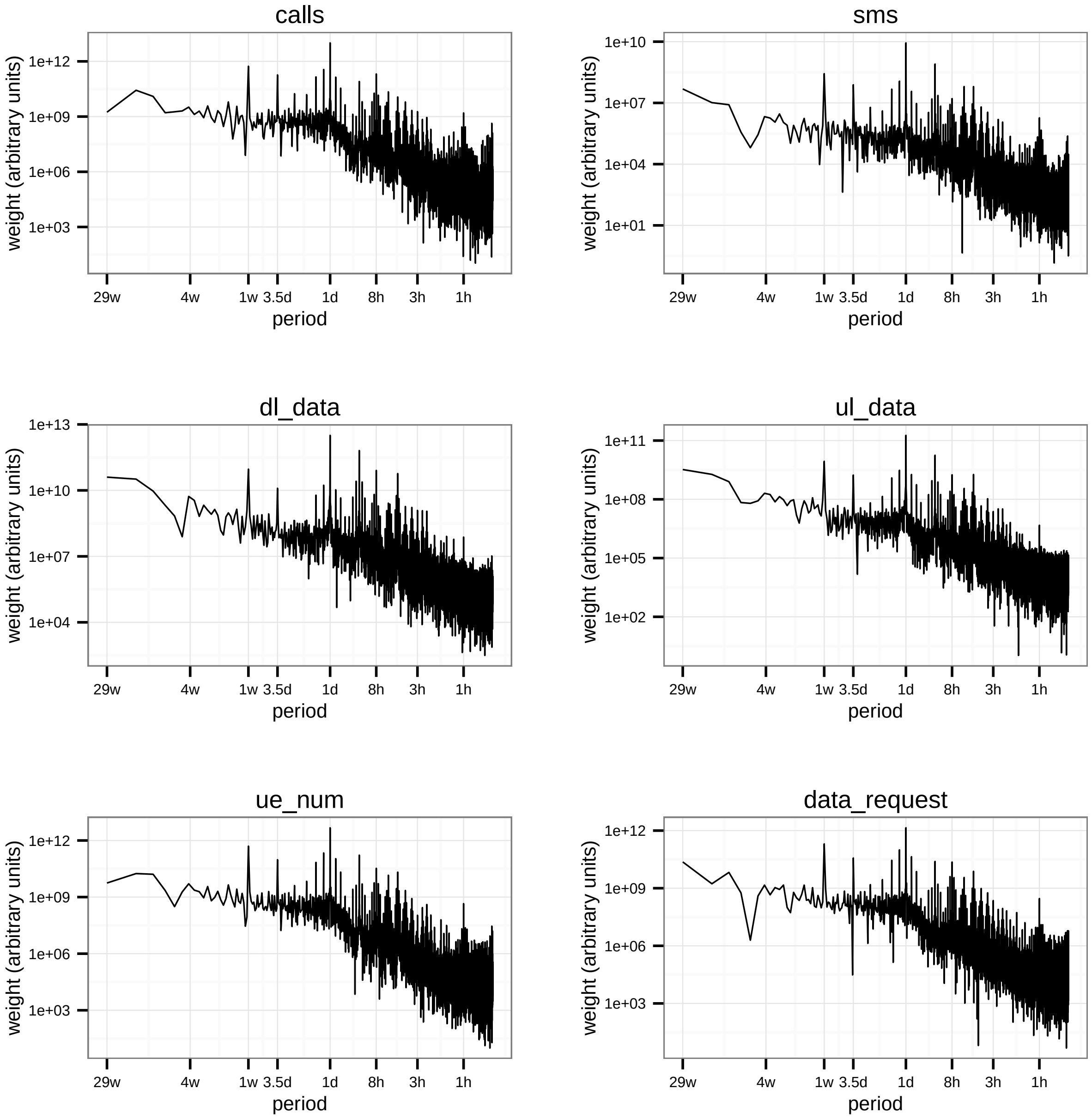

Figure S1: Power spectrum of time series. Note that the axes are on a logarithmic scale (and the x-axis displays the period of the component, with abbreviatoin of units, i.e. 'w' for week, 'd' for day and 'h' for hour). Power spectra were calculated for the interval between April 1st and October 20th 2013 to avoid artifacts due to significant amount of missing data on October 21th and the effects of DST change a week later.

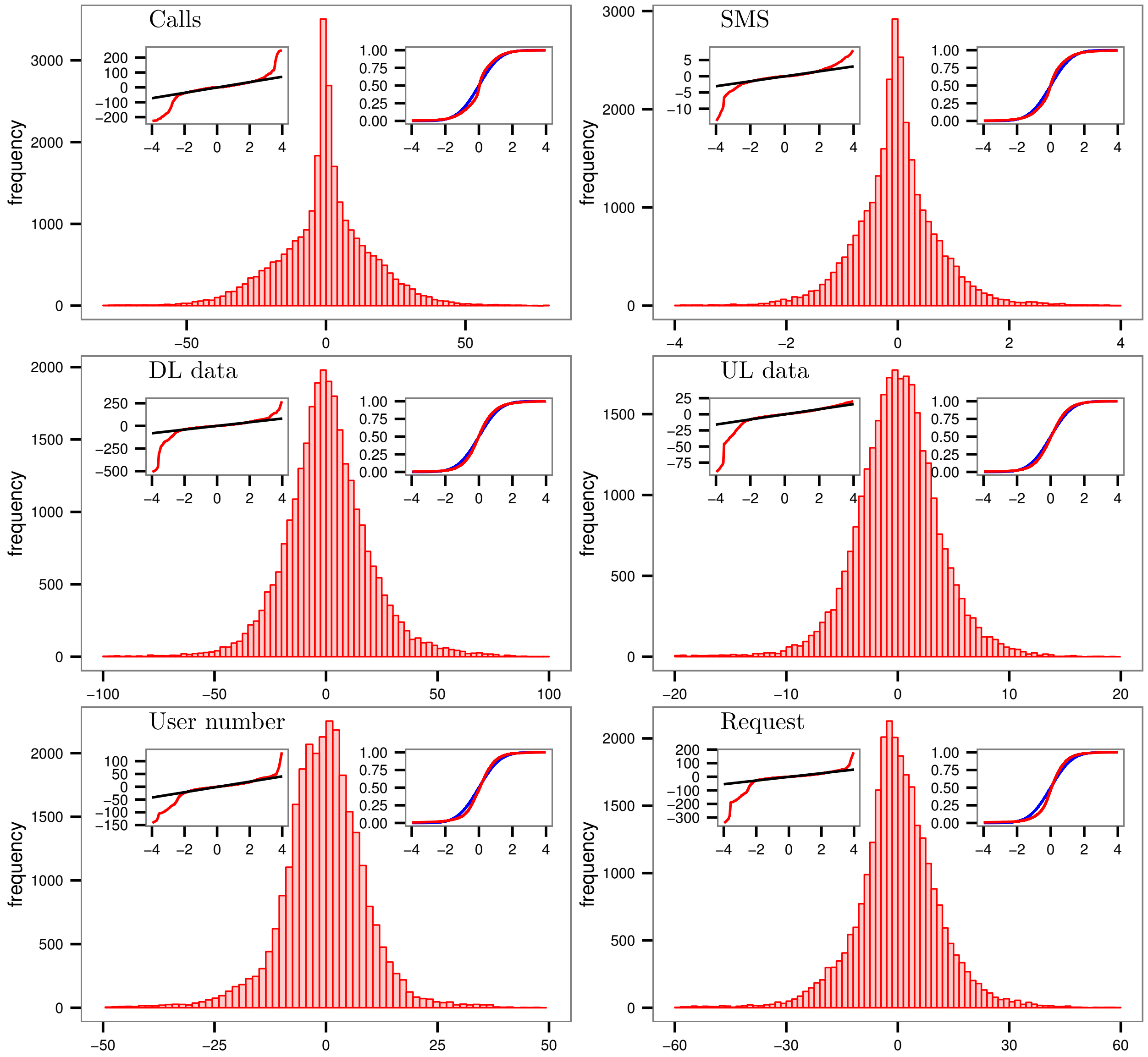

Figure S2: Distribution of (absolute) errors for all data types (whole city timelines, typical week with trends model). The main figures display the PDF of residuals, while the insets show tests for normal distribution. The left insets display quantile-quantile plots, with a linear fit to find the best parameters of the distribution. The right insets show the CDF as a function of standardized values compared with the CDF of a standard normal distribution. The largest difference of the two is used for performing the Kolmogorov-Smirnov test for normality. For all data types, the mid-part can be relatively well approximated by a normal distribution, with the tails considered outliers. Neverthelesss, the Kolmogorov-Smirnov test rejects the hypothesis of normal distribution in all cases. Further deviation is present in the case of calls and SMS around zero (as can be noticed in the spike in the PDF and in the CDF plots, while for the other data types, the distribution is somewhat skewed. This results in that the Kolmogorov-Smirnov test rejects the hypothesis of normal distribution in all cases.

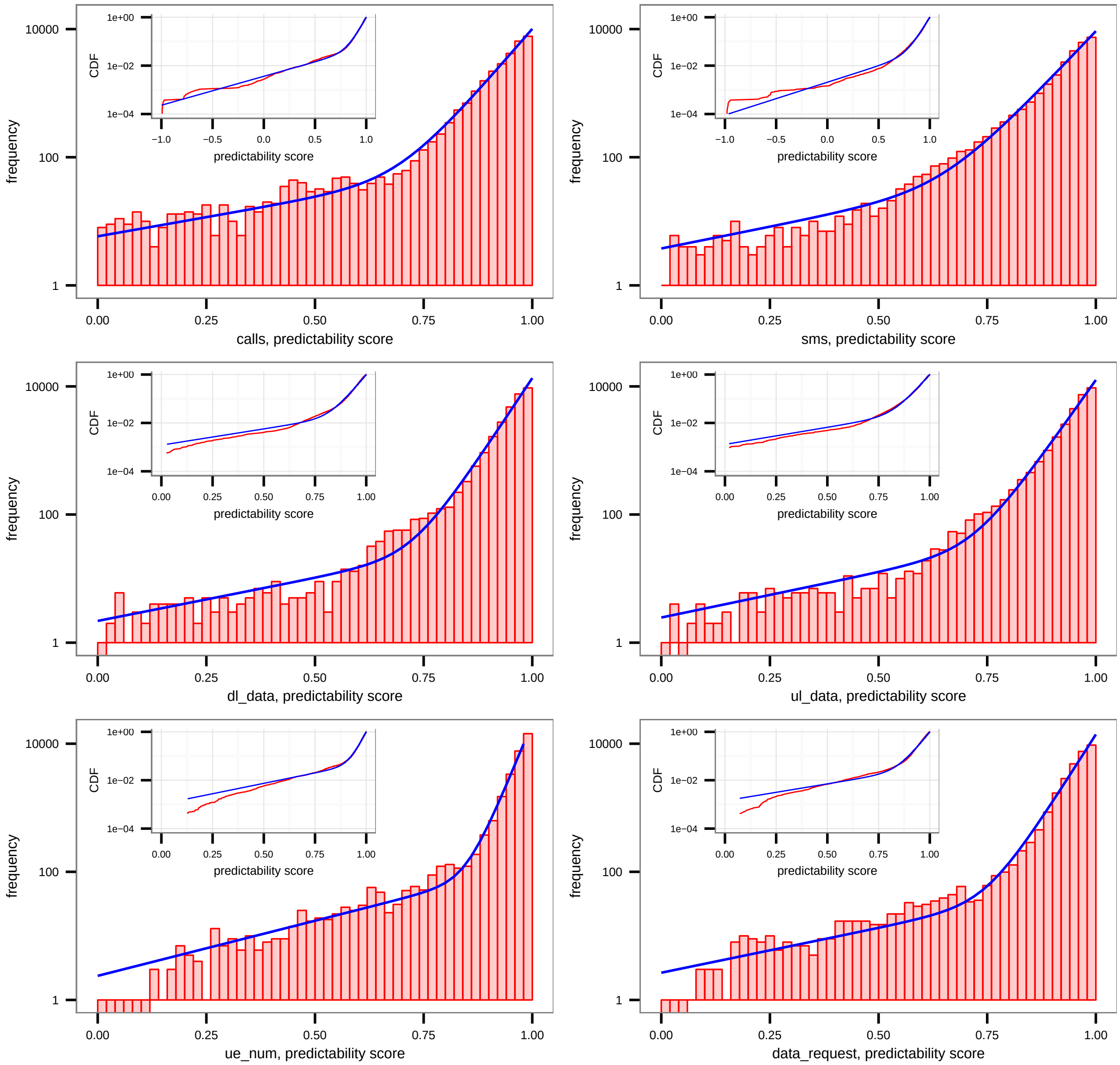

Figure S3: Distribution of predictability scores for all data types (predictability calculated for whole city timelines). All distibutions can be approximated quite well by the combination of two exponential functions: $P(\Phi) = A\lambda_1 e^{-\lambda_1(1-\Phi)} + (1-A)\lambda_2 e^{-\lambda_2(1-\Phi)}$ (note that the displayed fits were carried out to the logarithms of CDF values, as displayed in the insets, while the PDFs are displayed together with the appropriately scaled distribution function in the main panels). This suggests an interpretation where the exponential function for high predictability scores describes the bulk of the distributions and the general high regularity of human activities, while lower predictability scores are "outliers" corresponding to special events not explained by random fluctuations, but whose amplitude also roughly follows an exponential distribution.

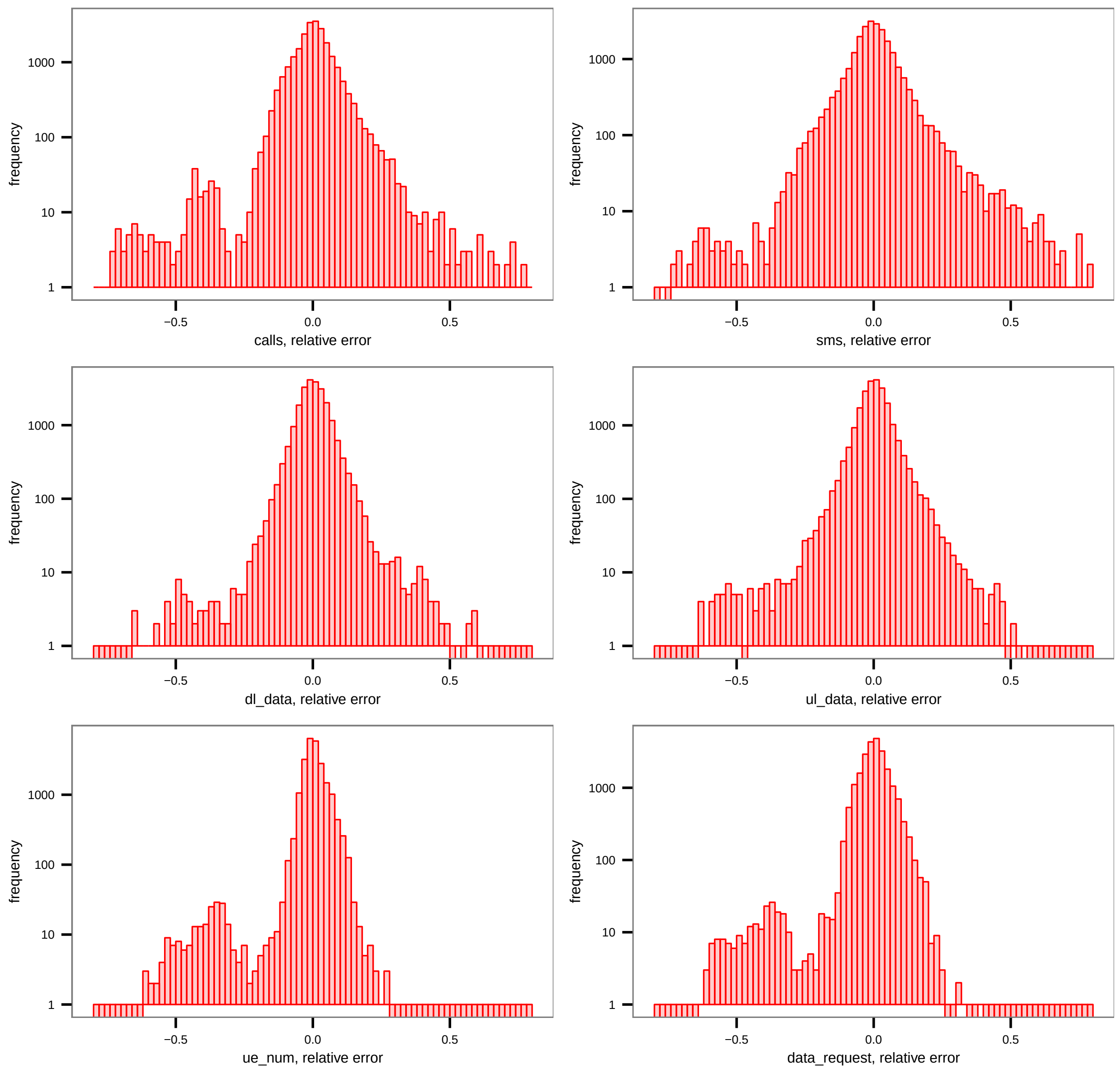

Figure S4: Distribution of relative errors for all data types (calculated for the whole city timeline). All distributions show exponential-like decrease around zero, indicating a general high regularity in the activities with outliers are again expected to correspond to special events or disruptions.

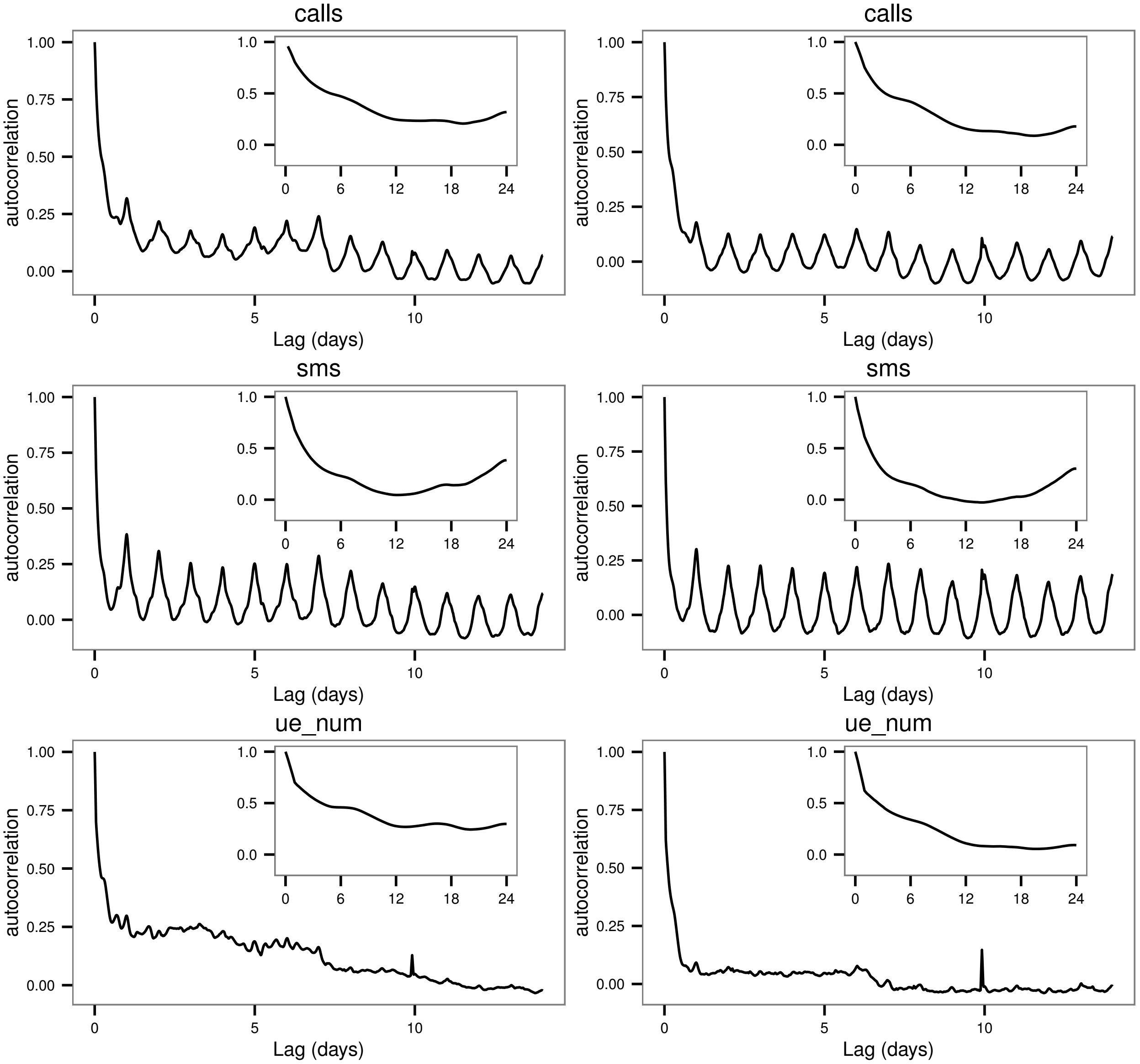

Figure S5: Temporal autocorrelation of predictability scores. Left column: whole period; right column: last two month including the Christmas season omitted. The insets show the autocorrelation function in the range of one day only. In all cases, autocorrelation shows a fast decrease, reaching almost zero in a few hours. Including the holiday season (left column) results in some correlation up to a few days, corresponding to the fact that around Christmas, activity patterns are significantly different, resulting in an extended period of time with low predictability (see also Fig. 1 in the main text for the effect of the holiday in the volume of activity). For most activity types (with the exception of user and data request numbers) a periodic fluctuation is present in the autocorrelation function; this can be explained by the fact that predictability correlates with activity volume which shows a strong periodic variation during the day.

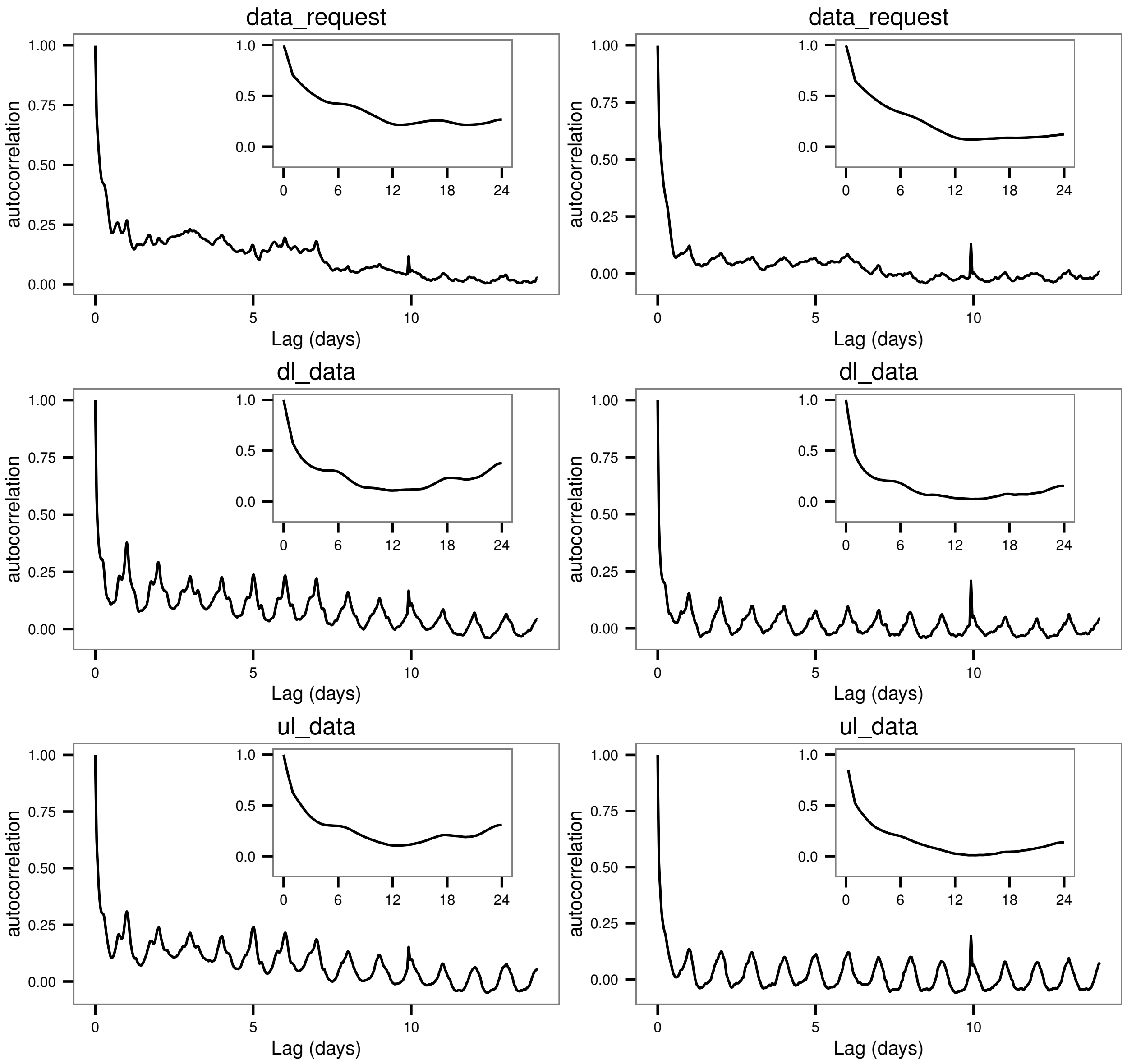

Figure S6: Temporal autocorrelation of predictability scores. Left column: whole period; right column: last two month including the Christmas season omitted. The insets show the autocorrelation function in the range of one day only.

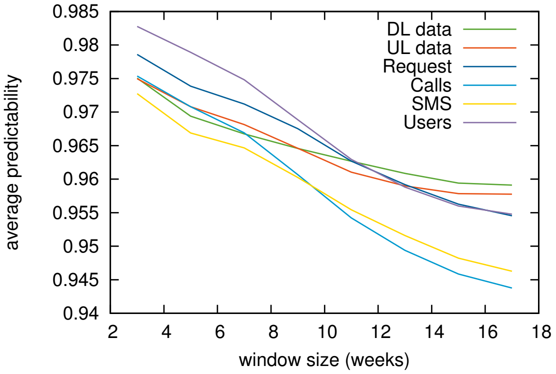

Figure S7: Sliding window average model, average predictability as a function of the window size used.

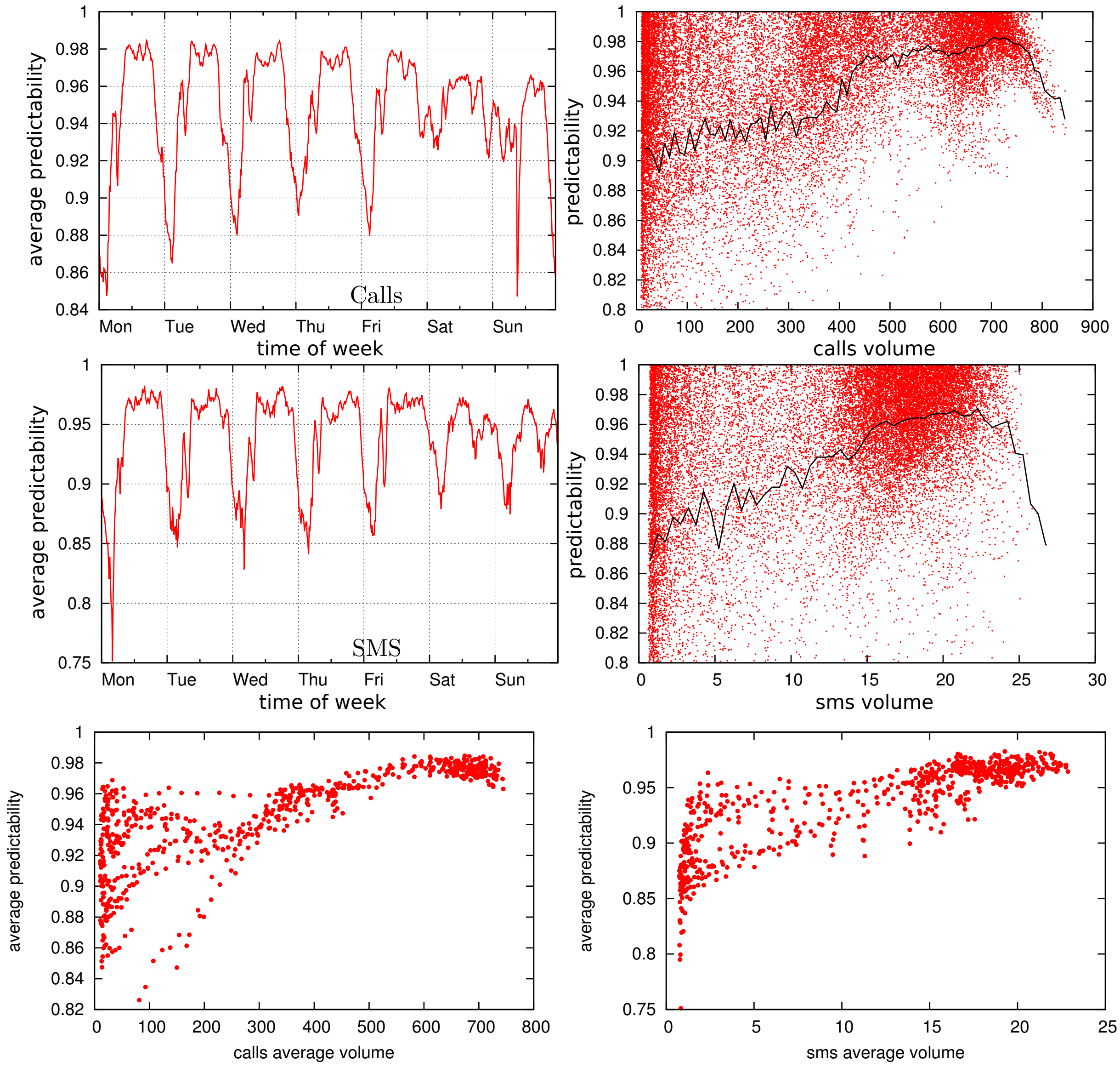

Figure S8: Top and middle row, left: average predictability values by the time of week for the whole city; right: city-wide predictability as a function of activity volume for each of the 15-min intervals in the measurement period. The black line is an average calculated as the function of volume. Top row: calls; middle row: SMS activity. The two datasets have a correlation coefficient of 0.293 for calls and 0.386 for SMS. Bottom row: average predictability for each 15-min interval in the week as a function of average volume (left: calls, right: SMS volume). Note that predictability scores display a daily period similarly to activity volumes. For most part, we see a slightly increasing trend, which can be thought of as the effect of the central limit theorem. For very high volumes however, predictability values are again smaller; we speculate that very high volumes correspond to special / one-time events which then represent a deviation from the normal rhythm of the city.

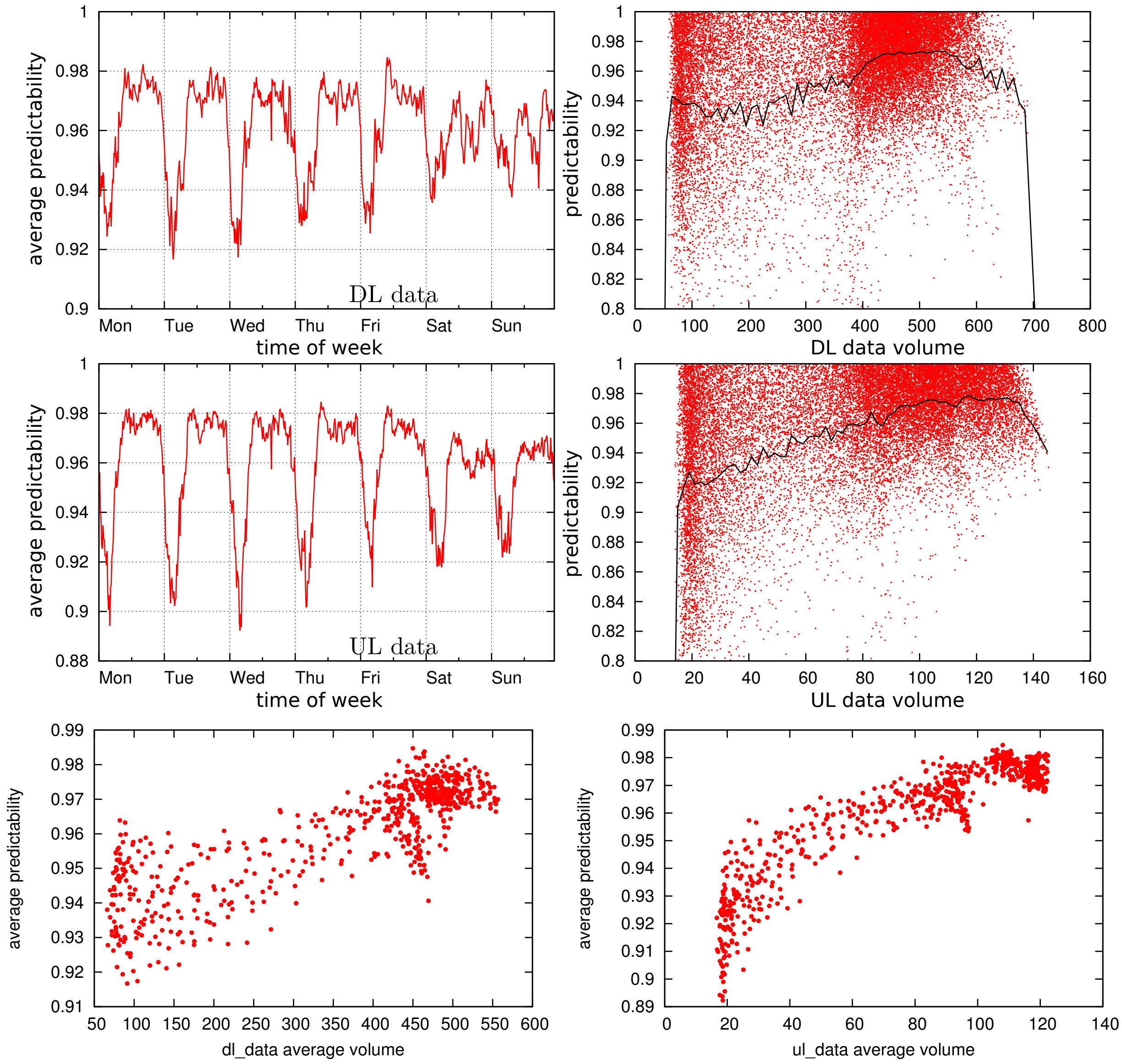

Figure S9: Top and middle row, left: average predictability values by the time of week for the whole city; right: city-wide predictability as a function of activity volume for each of the 15-min intervals in the measurement period. The black line is an average calculated as the function of volume. Top row: DL data; middle row: UL data traffic volume. The two datasets have a correlation coefficient of 0.256 for DL and 0.331 for UL data traffic activity. Bottom row: average predictability for each 15-min interval in the week as a function of average volume (left: DL data, right: UL data). Note that predictability scores display a daily period similarly to activity volumes. For most part, we see a slightly increasing trend, which can be thought of as the effect of the central limit theorem. Similarly to calls and SMS, predictability values are again smaller for very high volumes.

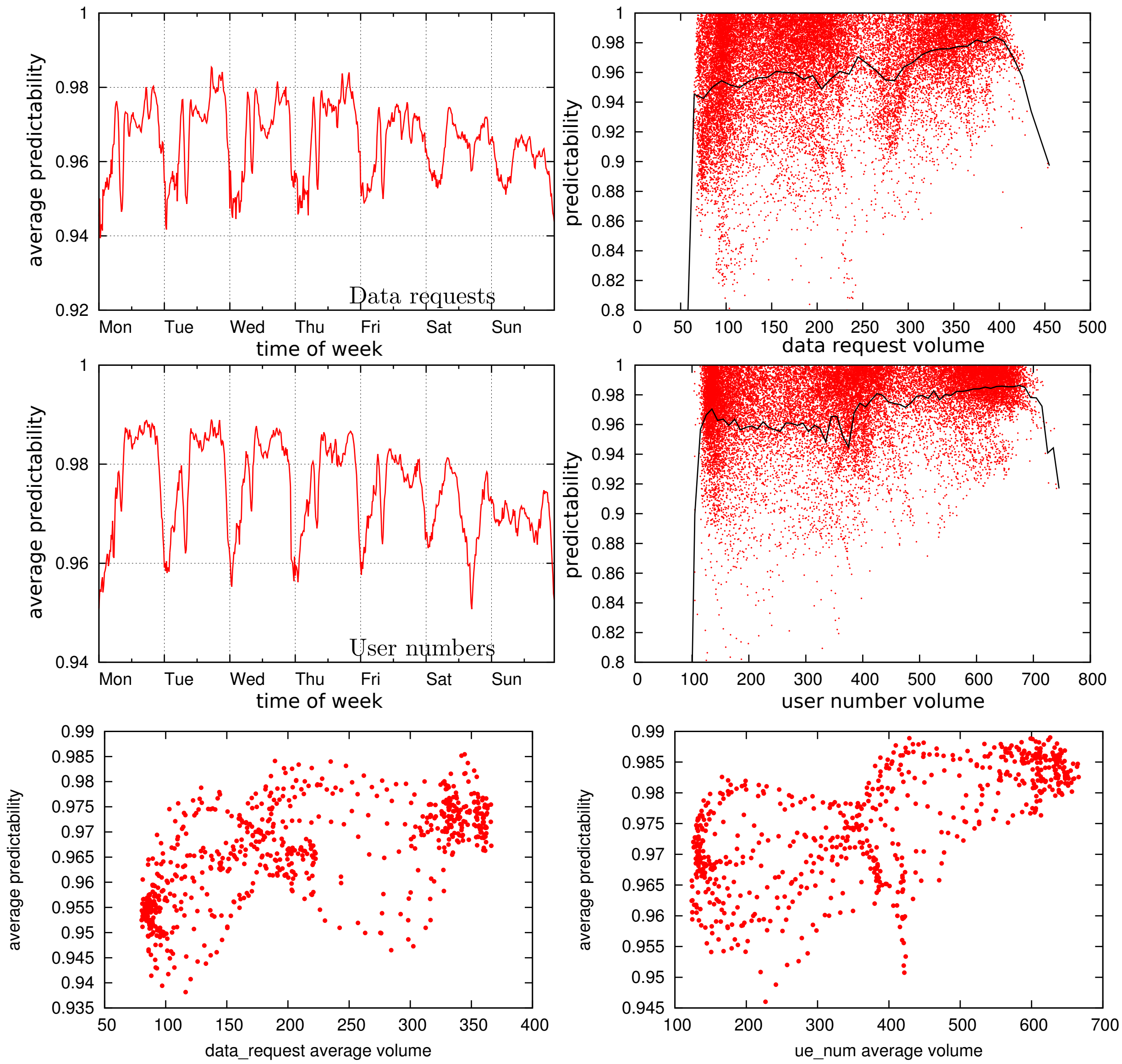

Figure S10: Top and middle row, left: average predictability values by the time of week for the whole city; right: city-wide predictability as a function of activity volume for each of the 15-min intervals in the measurement period. The black line is an average calculated as the function of volume. Top row: data request number; middle row: user number. The two datasets have a correlation coefficient of 0.159 for data requests and 0.158 for user numbers. Bottom row: average predictability for each 15-min interval in the week as a function of average volume (left: data requests, right: user number). Here, the overall trend is much less pronounced than for other activity types (note that the y-axis for predictability scores spans a much smaller range than in the case of other activity types).

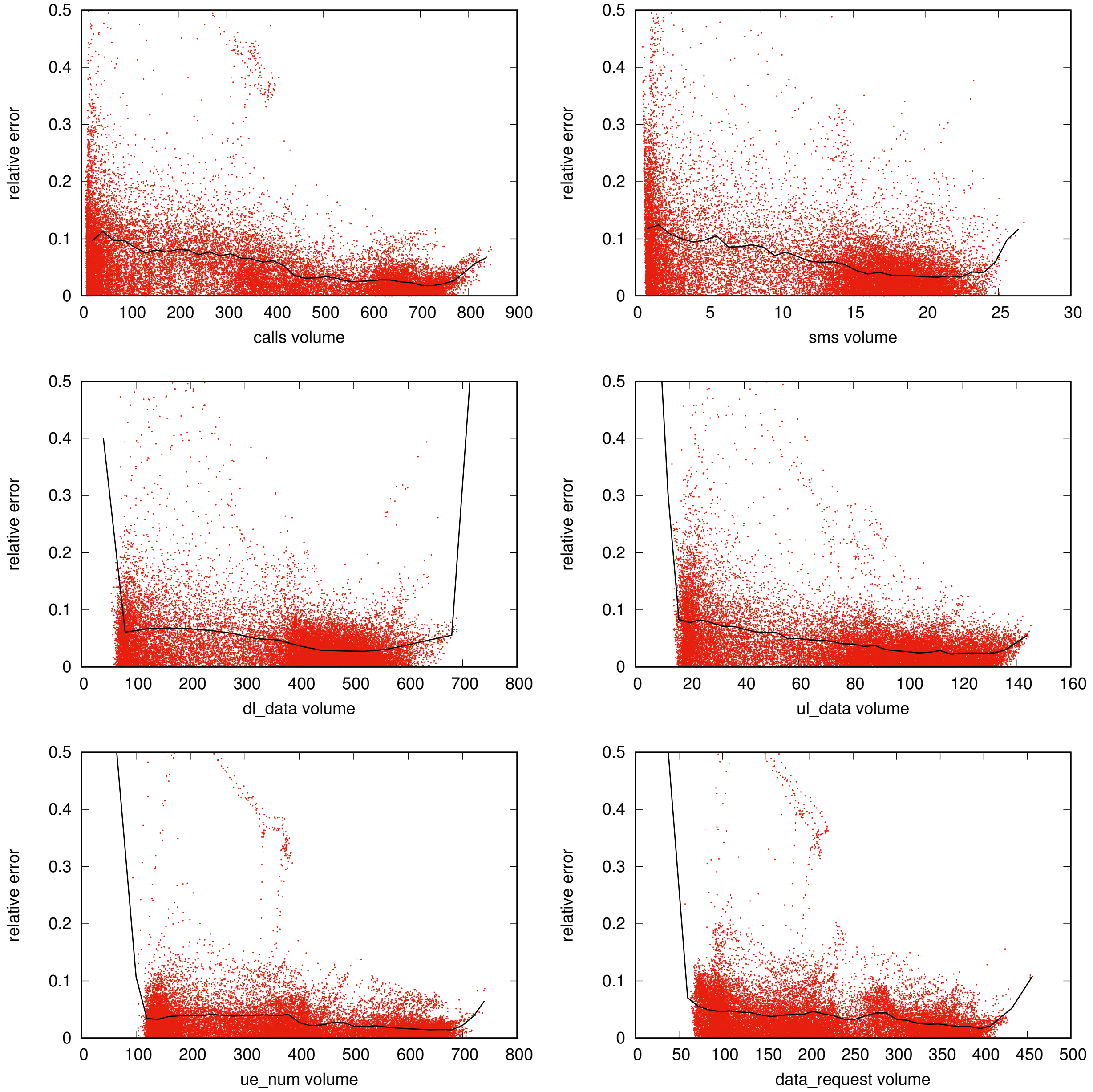

Figure S11: City-wide relative error (absolute values) as a function of activity volume in each of the 15-min intervals in the measurement period. The black line is an average calculated as the function of volume. We see that relative errors decrease with activity volume, but there is a significant scatter around this trend. Also, higher relative errors for very high activity volumes can be explained that these are outliers, i.e. these are not expected based on the typical activities, hence the higher deviations from them.

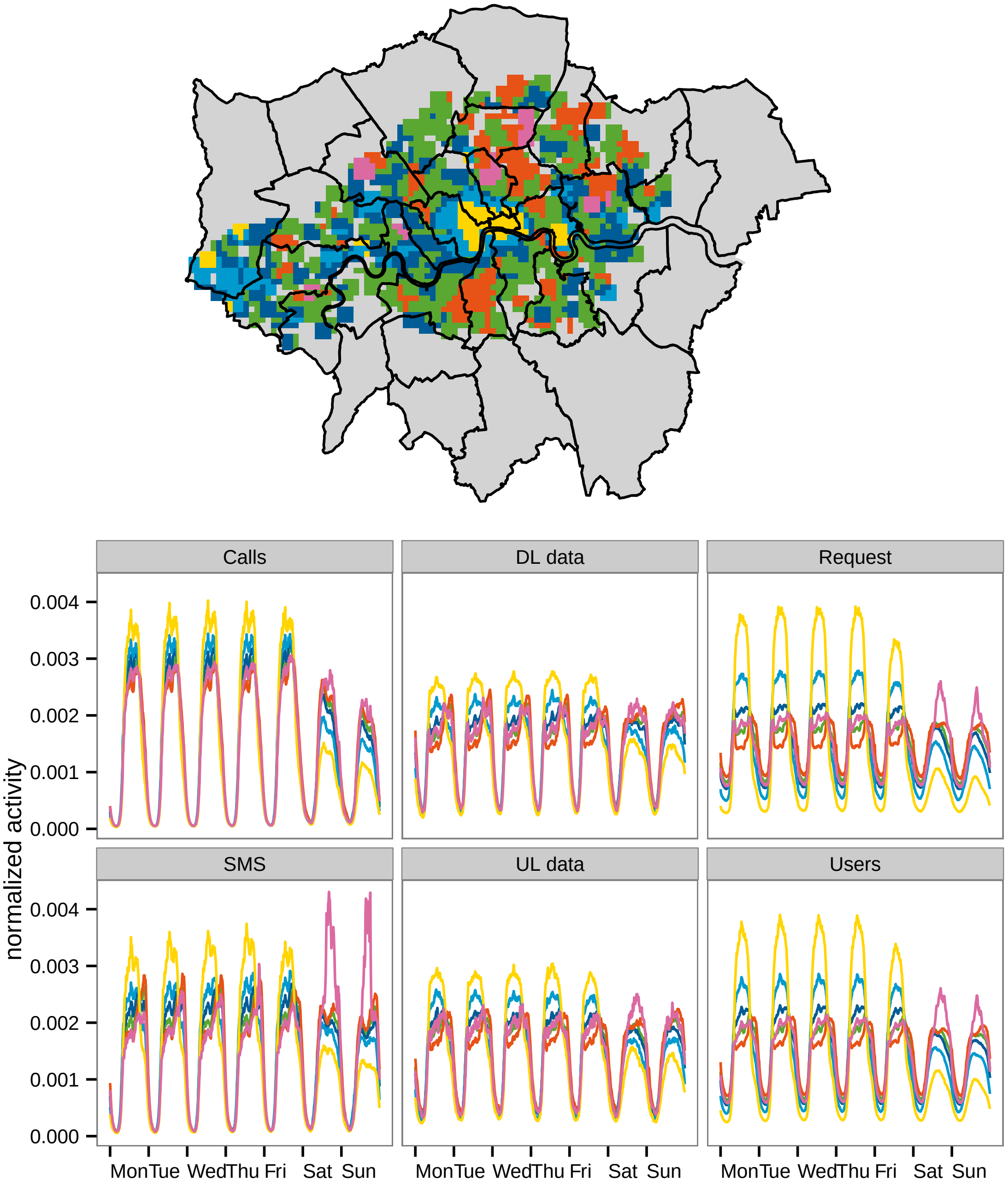

Figure S12: Functional clusters detected in the Greater London area based on normalized mobile network activity time series. We use the typical week of activities to cluster pixels with the k-means algorithm. The resulting spatial distribution is displayed in the top panel while cluster-wide average time series is displayed in the bottom panel. The clusters can be identified as core business, commercial, mixed (commercial / residential), residential, commuter and residential / leisure. The methodology and interpretation of clusters was discussed in more detail in [1].
Map was generated with `R`, using the `ggplot2`, `sp` and `rgdal` libraries (`https://www.r-project.org`, see also Ref. [44] in the main text); the background map was generated from the digital maps (shapefiles) available from the Greater London Authority (`http://data.london.gov.uk/dataset/statistical-gis-boundary-files-london`).

Predictability scores

	predictability at various spatial resolution					predictability of pixels using data from larger spatial aggregation			
Type	pixels	wards	boroughs	clusters	city	wards	boroughs	clusters	city
weighted averages									
DL data	0.811 (0.188)	0.794 (0.210)	0.925 (0.073)	0.953 (0.041)	0.966 (0.032)	0.789 (0.205)	0.775 (0.208)	0.791 (0.203)	0.754 (0.216)
UL data	0.76 (0.246)	0.740 (0.267)	0.900 (0.104)	0.946 (0.053)	0.968 (0.030)	0.738 (0.261)	0.727 (0.265)	0.739 (0.263)	0.707 (0.271)
Request	0.898 (0.110)	0.891 (0.121)	0.942 (0.059)	0.951 (0.039)	0.971 (0.026)	0.842 (0.151)	0.808 (0.165)	0.851 (0.140)	0.738 (0.194)
Calls	0.91 (0.100)	0.904 (0.112)	0.953 (0.051)	0.956 (0.040)	0.968 (0.035)	0.887 (0.123)	0.872 (0.128)	0.884 (0.127)	0.850 (0.146)
SMS	0.824 (0.200)	0.812 (0.214)	0.914 (0.105)	0.938 (0.076)	0.958 (0.043)	0.801 (0.218)	0.788 (0.226)	0.802 (0.222)	0.763 (0.236)
Users	0.927 (0.085)	0.922 (0.095)	0.961 (0.041)	0.963 (0.032)	0.980 (0.019)	0.880 (0.125)	0.843 (0.140)	0.888 (0.113)	0.787 (0.168)
unweighted averages									
DL data	0.687 (0.311)	0.579 (0.510)	0.817 (0.378)	0.939 (0.079)	0.959 (0.063)	0.671 (0.317)	0.663 (0.319)	0.667 (0.322)	0.654 (0.320)
UL data	0.665 (0.300)	0.578 (0.488)	0.793 (0.367)	0.923 (0.010)	0.958 (0.065)	0.650 (0.307)	0.641 (0.308)	0.643 (0.308)	0.639 (0.309)
Request	0.859 (0.188)	0.757 (0.450)	0.860 (0.363)	0.947 (0.073)	0.967 (0.059)	0.819 (0.209)	0.792 (0.215)	0.819 (0.204)	0.734 (0.230)
Calls	0.775 (0.322)	0.667 (0.529)	0.860 (0.336)	0.938 (0.082)	0.949 (0.073)	0.751 (0.329)	0.738 (0.333)	0.734 (0.344)	0.718 (0.341)
SMS	0.658 (0.406)	0.558 (0.575)	0.820 (0.338)	0.900 (0.134)	0.938 (0.083)	0.640 (0.411)	0.634 (0.411)	0.636 (0.415)	0.623 (0.411)
Users	0.898 (0.167)	0.796 (0.442)	0.895 (0.324)	0.960 (0.069)	0.976 (0.056)	0.860 (0.187)	0.834 (0.191)	0.866 (0.180)	0.783 (0.201)

Relative deviations

	predictability at various spatial resolution					predictability of pixels using data from larger spatial aggregation			
Type	pixels	wards	boroughs	clusters	city	wards	boroughs	clusters	city
weighted averages									
DL data	0.213 (0.350)	0.248 (0.511)	0.077 (0.159)	0.043 (0.043)	0.034 (0.032)	0.242 (0.402)	0.258 (0.418)	0.242 (0.408)	0.285 (0.444)
UL data	0.314 (0.658)	0.362 (0.824)	0.106 (0.160)	0.049 (0.064)	0.032 (0.030)	0.353 (0.816)	0.374 (0.916)	0.361 (0.898)	0.405 (0.994)
Request	0.106 (0.153)	0.116 (0.220)	0.058 (0.130)	0.041 (0.042)	0.029 (0.026)	0.167 (0.196)	0.204 (0.224)	0.159 (0.200)	0.286 (0.272)
Calls	0.092 (0.145)	0.102 (0.265)	0.048 (0.195)	0.036 (0.046)	0.032 (0.039)	0.118 (0.172)	0.134 (0.186)	0.123 (0.191)	0.159 (0.223)
SMS	0.217 (0.549)	0.240 (0.672)	0.094 (0.255)	0.059 (0.118)	0.043 (0.045)	0.256 (0.759)	0.293 (1.131)	0.275 (1.116)	0.334 (1.292)
Users	0.075 (0.122)	0.083 (0.156)	0.039 (0.051)	0.030 (0.037)	0.020 (0.019)	0.126 (0.164)	0.164 (0.187)	0.117 (0.161)	0.227 (0.230)
unweighted averages									
DL data	0.311 (0.415)	0.381 (1.202)	0.170 (2.319)	0.060 (0.068)	0.040 (0.044)	0.333 (0.465)	0.338 (0.468)	0.331 (0.457)	0.352 (0.490)
UL data	0.350 (0.536)	0.400 (1.111)	0.196 (1.549)	0.078 (0.104)	0.041 (0.047)	0.374 (0.638)	0.381 (0.675)	0.376 (0.657)	0.393 (0.730)
Request	0.137 (0.175)	0.202 (1.654)	0.153 (5.557)	0.053 (0.064)	0.033 (0.038)	0.178 (0.208)	0.206 (0.214)	0.179 (0.203)	0.274 (0.252)
Calls	0.212 (0.321)	0.280 (1.698)	0.143 (5.649)	0.062 (0.076)	0.051 (0.063)	0.241 (0.355)	0.250 (0.345)	0.249 (0.343)	0.273 (0.362)
SMS	0.329 (0.539)	0.392 (2.218)	0.183 (5.924)	0.103 (0.174)	0.062 (0.074)	0.356 (0.668)	0.362 (0.800)	0.355 (0.795)	0.381 (0.862)
Users	0.098 (0.135)	0.160 (0.587)	0.089 (0.942)	0.039 (0.060)	0.023 (0.033)	0.138 (0.175)	0.163 (0.174)	0.130 (0.159)	0.220 (0.196)

Table S2: Predictability scores and relative errors at various spatial resolutions, calculated using weighted averages and simple averages. The values in parentheses indicate the standard deviations of the respective values.

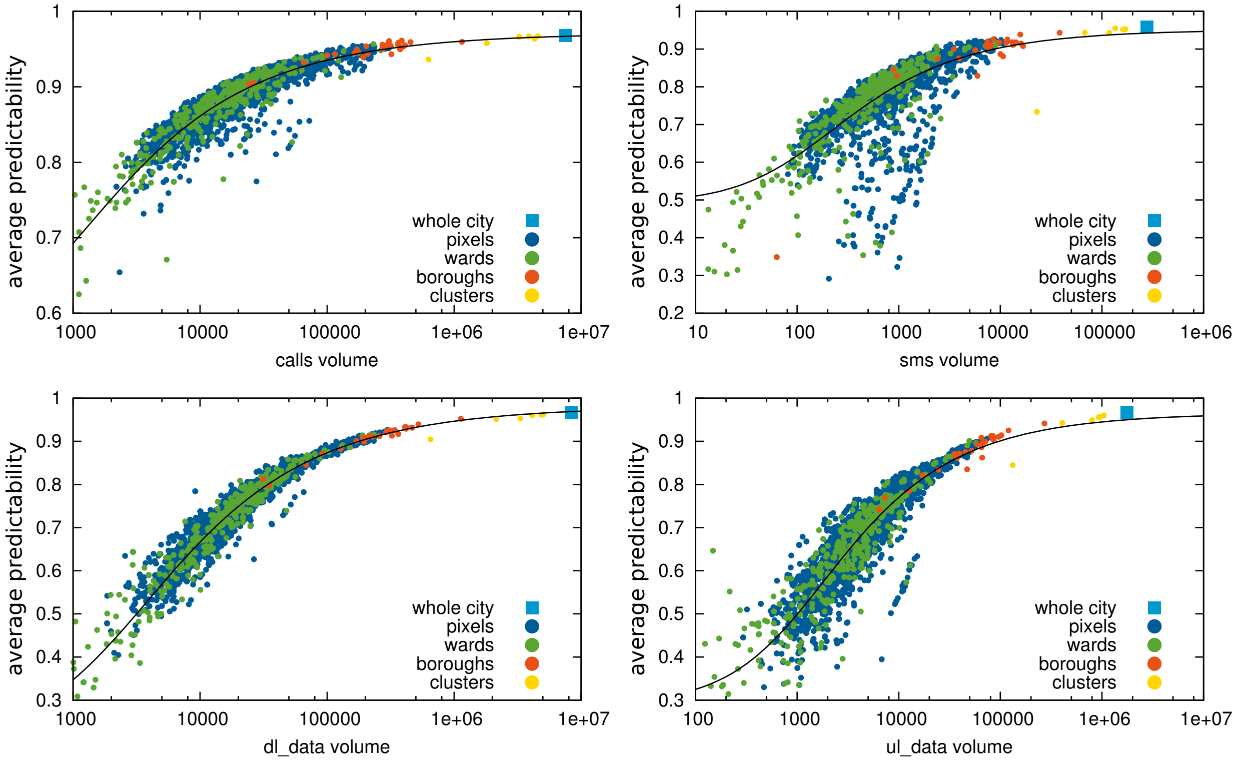

Figure S13: Average predictability scores at different spatial aggregation levels as a function of activity volume for Calls, SMS (top row), DL data and UL data traffic (bottom row). In each case, the empirical function $\Phi = 1 - \Delta - a/\sqrt{A+b}$ approximates the trend well. Values of the Δ empirical threshold for maximum predictability are $\Delta^{(\text{Calls})} = 0.029$, $\Delta^{(\text{SMS})} = 0.049$, $\Delta^{(\text{DL data})} = 0.019$ and $\Delta^{(\text{UL data})} = 0.035$.

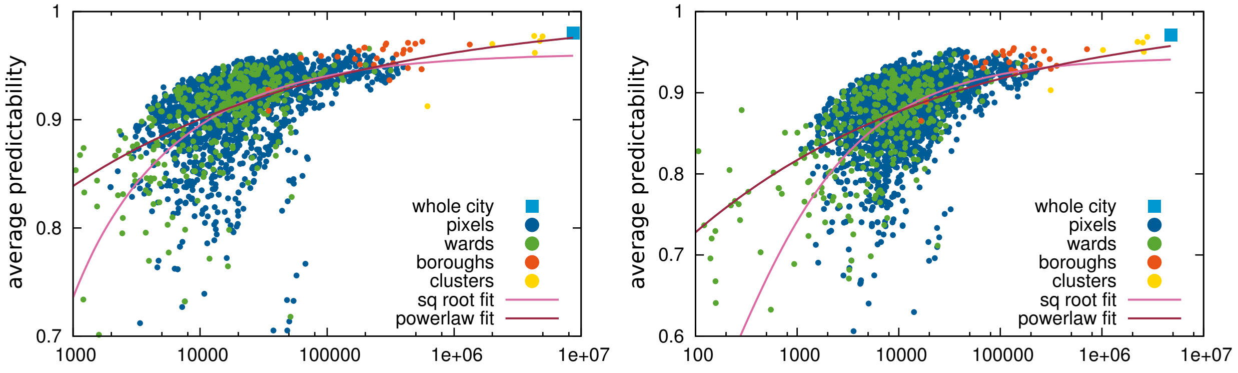

Figure S14: Average predictability scores at different spatial aggregation levels as a function of activity volume for data reuqest and user numbers. Here, while the predictability scores are higher in general, the dependence on the average volume is less pronounced, and fitting it with a plausible functional form is more challenging. In contrast to the previous functional form which misses the data points corresponding to high-volume regions and thus approximates the possible Δ threshold value, power-law fits in the form of $\Phi = 1 - aA^{-\gamma}$ with $\gamma^{(\text{Request})} = 0.17$ and $\gamma^{(\text{Users})} = 0.21$ seems to give better results, although it is not very convincing either, given the high variance in predictability scores in this case.

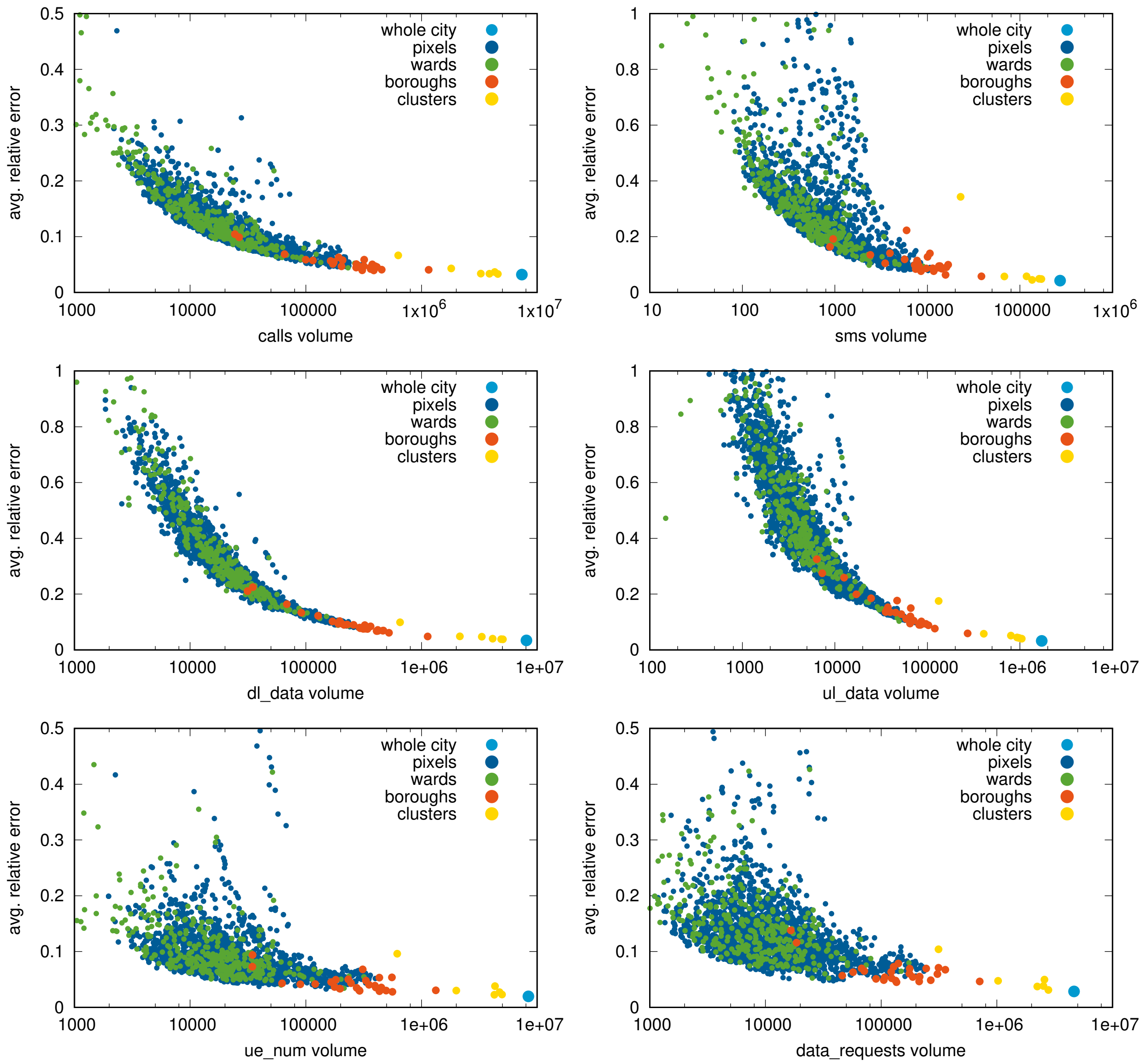

Figure S15: Average relative error as a function of volume for all spatial aggregation units and data types.

cluster	DL data	UL ata	Request	Calls	SMS	Users
weighted averages						
residential	0.962 (0.036)	0.957 (0.042)	0.962 (0.029)	0.964 (0.038)	0.952 (0.051)	0.973 (0.022)
mixed	0.962 (0.035)	0.960 (0.037)	0.969 (0.027)	0.967 (0.036)	0.952 (0.052)	0.977 (0.021)
commuter	0.952 (0.044)	0.943 (0.051)	0.953 (0.035)	0.958 (0.047)	0.943 (0.057)	0.970 (0.023)
commercial	0.960 (0.036)	0.956 (0.042)	0.963 (0.034)	0.967 (0.036)	0.955 (0.042)	0.977 (0.020)
core business	0.953 (0.041)	0.949 (0.048)	0.950 (0.044)	0.966 (0.035)	0.943 (0.052)	0.962 (0.032)
residential / leisure	0.905 (0.089)	0.845 (0.160)	0.903 (0.101)	0.936 (0.076)	0.734 (0.259)	0.912 (0.118)
unweighted averages						
residential	0.954 (0.067)	0.945 (0.075)	0.957 (0.061)	0.944 (0.076)	0.924 (0.098)	0.970 (0.057)
mixed	0.954 (0.066)	0.948 (0.071)	0.964 (0.059)	0.947 (0.075)	0.925 (0.100)	0.973 (0.057)
commuter	0.942 (0.074)	0.931 (0.082)	0.948 (0.063)	0.937 (0.083)	0.921 (0.096)	0.968 (0.057)
commercial	0.951 (0.067)	0.941 (0.076)	0.958 (0.062)	0.942 (0.081)	0.933 (0.085)	0.973 (0.057)
core business	0.941 (0.073)	0.927 (0.088)	0.940 (0.072)	0.945 (0.075)	0.919 (0.092)	0.950 (0.064)
residential / leisure	0.895 (0.105)	0.845 (0.146)	0.914 (0.101)	0.917 (0.098)	0.775 (0.212)	0.929 (0.102)

Table S3: Predictability scores of the individual clusters.

cluster	DL data	UL ata	Request	Calls	SMS	Users
weighted averages						
residential	0.039 (0.037)	0.043 (0.044)	0.038 (0.030)	0.037 (0.044)	0.049 (0.055)	0.027 (0.022)
mixed	0.038 (0.035)	0.040 (0.038)	0.031 (0.027)	0.033 (0.040)	0.049 (0.062)	0.023 (0.021)
commuter	0.049 (0.046)	0.058 (0.054)	0.047 (0.035)	0.043 (0.066)	0.058 (0.066)	0.030 (0.023)
commercial	0.040 (0.037)	0.044 (0.043)	0.036 (0.034)	0.033 (0.039)	0.045 (0.043)	0.022 (0.020)
core business	0.048 (0.042)	0.052 (0.052)	0.048 (0.044)	0.034 (0.037)	0.058 (0.055)	0.038 (0.031)
residential / leisure	0.100 (0.107)	0.177 (0.247)	0.105 (0.142)	0.067 (0.095)	0.348 (0.540)	0.097 (0.160)
unweighted averages						
residential	0.045 (0.050)	0.055 (0.062)	0.042 (0.041)	0.056 (0.067)	0.076 (0.095)	0.030 (0.035)
mixed	0.045 (0.049)	0.051 (0.056)	0.035 (0.039)	0.053 (0.065)	0.075 (0.110)	0.027 (0.035)
commuter	0.057 (0.060)	0.069 (0.071)	0.051 (0.045)	0.063 (0.080)	0.079 (0.095)	0.031 (0.035)
commercial	0.048 (0.051)	0.058 (0.063)	0.042 (0.044)	0.058 (0.072)	0.067 (0.074)	0.026 (0.034)
core business	0.058 (0.058)	0.073 (0.082)	0.060 (0.057)	0.054 (0.064)	0.081 (0.084)	0.049 (0.045)
residential / leisure	0.106 (0.104)	0.161 (0.183)	0.088 (0.110)	0.084 (0.099)	0.238 (0.342)	0.073 (0.114)

Table S4: Relative errors in the individual clusters

cluster	DL data	UL ata	Request	Calls	SMS	Users
weighted averages						
residential	0.776 (0.209)	0.704 (0.276)	0.889 (0.116)	0.897 (0.110)	0.804 (0.208)	0.921 (0.088)
mixed	0.802 (0.191)	0.742 (0.250)	0.894 (0.108)	0.906 (0.102)	0.817 (0.202)	0.923 (0.084)
commuter	0.755 (0.222)	0.686 (0.288)	0.880 (0.122)	0.889 (0.119)	0.800 (0.204)	0.920 (0.090)
commercial	0.857 (0.147)	0.813 (0.194)	0.908 (0.098)	0.925 (0.082)	0.860 (0.153)	0.940 (0.063)
core business	0.895 (0.106)	0.867 (0.144)	0.922 (0.079)	0.942 (0.062)	0.891 (0.119)	0.944 (0.054)
residential / leisure	0.757 (0.221)	0.656 (0.312)	0.824 (0.228)	0.875 (0.153)	0.584 (0.390)	0.852 (0.223)
unweighted averages						
residential	0.673 (0.313)	0.654 (0.301)	0.859 (0.184)	0.764 (0.333)	0.645 (0.416)	0.899 (0.162)
mixed	0.689 (0.315)	0.665 (0.305)	0.859 (0.198)	0.772 (0.329)	0.660 (0.407)	0.894 (0.181)
commuter	0.650 (0.331)	0.640 (0.312)	0.849 (0.205)	0.756 (0.343)	0.636 (0.423)	0.893 (0.184)
commercial	0.736 (0.282)	0.708 (0.277)	0.868 (0.166)	0.808 (0.277)	0.705 (0.369)	0.908 (0.142)
core business	0.803 (0.231)	0.761 (0.247)	0.877 (0.139)	0.851 (0.225)	0.768 (0.305)	0.911 (0.110)
residential / leisure	0.703 (0.270)	0.659 (0.277)	0.838 (0.177)	0.805 (0.238)	0.602 (0.369)	0.882 (0.154)

Table S5: Average pixel-wide predictability scores in the individual clusters.

cluster	DL data	UL ata	Request	Calls	SMS	Users
			weighted averages			
residential	0.265 (0.419)	0.412 (0.807)	0.116 (0.151)	0.108 (0.160)	0.246 (0.603)	0.081 (0.111)
mixed	0.228 (0.354)	0.344 (0.692)	0.110 (0.135)	0.098 (0.139)	0.230 (0.602)	0.079 (0.103)
commuter	0.294 (0.454)	0.449 (0.861)	0.127 (0.159)	0.118 (0.198)	0.246 (0.550)	0.083 (0.115)
commercial	0.156 (0.238)	0.224 (0.445)	0.096 (0.121)	0.077 (0.105)	0.157 (0.291)	0.061 (0.071)
core business	0.110 (0.151)	0.149 (0.268)	0.080 (0.089)	0.058 (0.073)	0.117 (0.189)	0.057 (0.058)
residential / leisure	0.291 (0.421)	0.506 (0.875)	0.233 (0.525)	0.143 (0.275)	0.736 (1.379)	0.199 (0.482)
			unweighted averages			
residential	0.325 (0.423)	0.363 (0.554)	0.136 (0.161)	0.222 (0.324)	0.340 (0.563)	0.097 (0.130)
mixed	0.307 (0.405)	0.348 (0.534)	0.135 (0.159)	0.213 (0.340)	0.326 (0.539)	0.099 (0.129)
commuter	0.347 (0.483)	0.377 (0.581)	0.146 (0.228)	0.229 (0.348)	0.348 (0.560)	0.102 (0.159)
commercial	0.261 (0.342)	0.302 (0.451)	0.129 (0.149)	0.183 (0.261)	0.285 (0.446)	0.089 (0.108)
core business	0.197 (0.286)	0.246 (0.385)	0.122 (0.137)	0.145 (0.224)	0.228 (0.378)	0.088 (0.096)
residential / leisure	0.298 (0.349)	0.361 (0.508)	0.166 (0.259)	0.192 (0.251)	0.405 (0.580)	0.121 (0.215)

Table S6: Average pixel-wide relative errors in the individual clusters.

cluster	DL data	UL ata	Request	Calls	SMS	Users
			weighted averages			
residential	0.762 (0.218)	0.689 (0.291)	0.858 (0.133)	0.879 (0.126)	0.791 (0.228)	0.900 (0.102)
mixed	0.788 (0.200)	0.727 (0.262)	0.855 (0.129)	0.886 (0.120)	0.801 (0.223)	0.897 (0.101)
commuter	0.743 (0.231)	0.673 (0.300)	0.841 (0.149)	0.873 (0.136)	0.786 (0.218)	0.895 (0.106)
commercial	0.827 (0.172)	0.786 (0.217)	0.854 (0.133)	0.886 (0.134)	0.824 (0.190)	0.892 (0.106)
core business	0.853 (0.132)	0.828 (0.168)	0.851 (0.138)	0.901 (0.105)	0.854 (0.145)	0.873 (0.115)
residential / leisure	0.740 (0.233)	0.636 (0.332)	0.765 (0.245)	0.849 (0.165)	0.555 (0.425)	0.802 (0.234)
			unweighted averages			
residential	0.658 (0.322)	0.635 (0.306)	0.831 (0.194)	0.733 (0.349)	0.630 (0.423)	0.878 (0.167)
mixed	0.671 (0.326)	0.646 (0.312)	0.824 (0.209)	0.733 (0.350)	0.641 (0.416)	0.869 (0.188)
commuter	0.635 (0.340)	0.626 (0.315)	0.818 (0.214)	0.727 (0.357)	0.621 (0.428)	0.873 (0.188)
commercial	0.695 (0.313)	0.662 (0.309)	0.790 (0.207)	0.720 (0.336)	0.650 (0.399)	0.837 (0.186)
core business	0.764 (0.245)	0.726 (0.259)	0.769 (0.209)	0.782 (0.263)	0.725 (0.322)	0.811 (0.174)
residential / leisure	0.686 (0.276)	0.644 (0.282)	0.786 (0.194)	0.774 (0.252)	0.590 (0.374)	0.832 (0.163)

Table S7: Average pixel-wide predictability scores in the individual clusters when using the clusters' typical week timelines to predict the pixels' activity.

cluster	DL data	UL ata	Request	Calls	SMS	Users
			weighted averages			
residential	0.287 (0.492)	0.463 (1.087)	0.151 (0.180)	0.129 (0.195)	0.303 (1.250)	0.105 (0.137)
mixed	0.248 (0.405)	0.385 (0.963)	0.154 (0.170)	0.120 (0.173)	0.290 (1.344)	0.107 (0.133)
commuter	0.314 (0.522)	0.499 (1.202)	0.171 (0.205)	0.138 (0.248)	0.291 (1.320)	0.109 (0.141)
commercial	0.192 (0.289)	0.266 (0.590)	0.154 (0.175)	0.123 (0.203)	0.210 (0.472)	0.112 (0.122)
core business	0.157 (0.207)	0.196 (0.400)	0.156 (0.167)	0.102 (0.128)	0.159 (0.255)	0.131 (0.128)
residential / leisure	0.321 (0.509)	0.589 (1.211)	0.316 (0.629)	0.173 (0.305)	0.986 (2.277)	0.268 (0.577)
			unweighted averages			
residential	0.342 (0.467)	0.386 (0.664)	0.164 (0.178)	0.249 (0.345)	0.359 (0.825)	0.117 (0.140)
mixed	0.324 (0.439)	0.372 (0.661)	0.170 (0.178)	0.248 (0.340)	0.351 (0.823)	0.125 (0.144)
commuter	0.361 (0.517)	0.397 (0.711)	0.178 (0.219)	0.253 (0.361)	0.369 (0.852)	0.122 (0.165)
commercial	0.301 (0.389)	0.347 (0.552)	0.217 (0.234)	0.266 (0.347)	0.340 (0.582)	0.163 (0.172)
core business	0.250 (0.399)	0.300 (0.578)	0.250 (0.281)	0.219 (0.298)	0.287 (0.513)	0.200 (0.208)
residential / leisure	0.320 (0.413)	0.388 (0.656)	0.221 (0.309)	0.226 (0.285)	0.424 (0.794)	0.174 (0.252)

Table S8: Average pixel-wide relaitve errors in the individual clusters when using the clusters' typical week timelines to predict the pixels' activity.

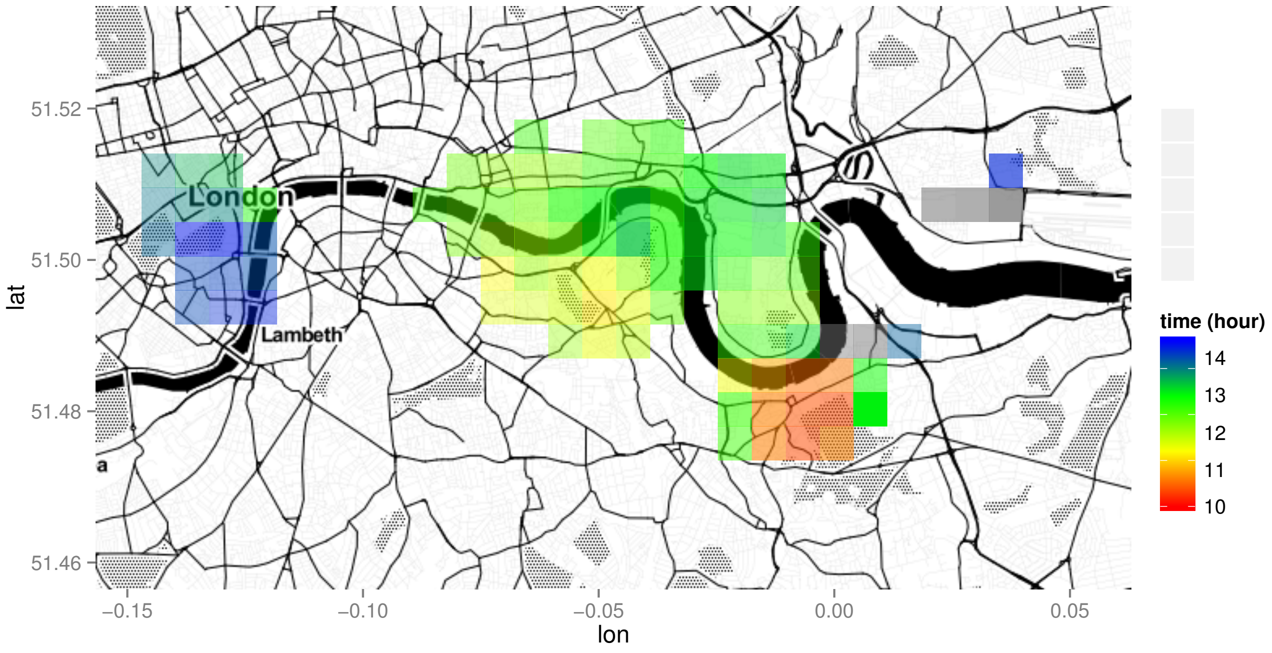

Figure S16: Outlier pixels found in central London on the 21st April, the day of the 2013 spring Marathon race. Opacity of the pixels correspond to the average predictability score for the day, with more opaque meaning less predictable in general, while color corresponds to the time interval with the least predictability during the day. The course of the race is followed by the outliers; the race started in Greenwich in the morning, than went west, then crossed the Thames and went back to the Docklands area, then headed to the Buckingham Palace where the end was. This is marked by the color of the pixels changing from red through yellow and green to blue.
Map was generated with `R`, using the `ggplot2` and `ggmap` libraries [?]; background map tiles by Stamen Design [?], under CC BY 3.0. Data by OpenStreetMap [?], under ODbL.

1 Notes on data processing

Data for this study was provided by a network operator with a significant market share in the London area and includes 3G traffic (as stated in the main text). The raw data was provided on the level of individual antennas and includes the number of calls, text messages and data communication requests, as well as the volume of uploaded and and downloaded data and unique users connected to the antenna in 15 minute intervals. All data was scaled with an unknown factor for confidentiality reasons. As the data contains only scaled aggregate counts and volumes, no identifiable information is provided about individual users or the type of the subscription (i.e. personal or business) or the content or purpose of the call or other communication. Spatial analysis is made avaiable by including the approximate locations of antennas as supplementary data to the measures. Due to the sensitive nature of the data, locations are only approximate and no information about the reception area of antennas was provided. This can range from around 100m to several kilometers, meaning that spatial aggregation will only be approximate. A phone will generally connect to the antenna with the highest signal level, suggesting an expectation of finding the closest antenna and thus using a Voronoi-tesselation as an approximation of the reception areas, several important factors affect this (network load, antenna visibility for the radio signal, variation in the configuration of antennas including direction, power, planned serving area), estimation of which were beyond the scope of our work, partly due to the sensitive nature of data about the details of the network configuration of the operator. To compensate for this, the main analysis is performed by aggregating antenna locations into an 500m by 500m regular grid ('pixels') and performing a further smoothing using a 3x3 neighborhood of each grid pixel. During our previous analysis using the same dataset [1], this kind of spatial aggregation was found to be sufficient to reveal a meaningful clustering of urban areas based on the activity time series. For the analysis based on administrative units (wards and boroughs), an antenna is assigned into the polygon which contains it's coordinates. While this introduces an uncertainty for antennas close to borders, we believe that the resulting activity time series are representative for the spatial aggregation on the given resolution and thus the calculated predictability measures are useful for characterizing the regularity of activities on the scale of wards and boroughs respectively.

We performed a comparison of the spatial distribution of activities to the population distribution as obtained from London mayor's office based on the last UK census [2]. We used the pycnophylactic interpolation methodolgy described in [3] to interpolate the data into the same 500m by 500m grid. A comparison based on these results is then presented in Fig. S17, while measures of correlation are displayed in Fig. S18. The maximum correlation values, achieved at late night are around 0.4 when considering the whole area, while they reach around 0.55 if we exclude the immediate city center which has relatively low population but a highly active nightlife and a high number of transient population, e.g. visitors staying in hotels who are not accounted for in the census data. While these values are significant, they are still relatively low. We note that this can be explained by the nature of the dataset: most importantly, mobile communications data only measures *active* population (while active can also mean a background process running on the phone requesting data communication over the cellular network). Apart form the ratio of people not staying home, a significant bias can result from different usage patterns when staying at home versus going out. Also, since many phones connect to a WiFi network when the user is at home, a significant ratio of data connections is not recorded in that case, further skewing the measures to overrepresent people staying out. Further differences are expected based on the uncertainty of the exact coverage areas of the antennas, especially at the border of the covered areas, which is a limitation of the current study, while more precise measurements implemented at the network operator level could improve this aspect. We expect that this has little effect on the evaluation of regularity and predictability of activities, where we expect that the typical aggregation of activities presented in this study is representative regarding the spatial resolutions employed.

Activity-based clustering was performed using the methodology described in Ref. [1]. The typical week time series for each pixel was obtained from the data after leaving out public holidays and outlier days. After that, a k-means clustering procedure was applied to the typical week time series; six clusters were chosen based on a local maximum in the silhouette index and good interpretability [1]. Resulting clusters and average time series are displayed in Fig. S12.

References

[1] Grauwin S, Sobolevsky S, Moritz S, Gódor I, Ratti C (2014). Towards a comparative science of cities: using mobile traffic records in New York, London and Hong Kong. In *Computational Approaches for Urban Environments* (eds. Helbich, M., Arsanjani, J. J. & Leitner M.), Ch. 15, 363–387 (Springer, 2014). Available at `http://arxiv.org/abs/1406.4400`.

[2] Population data based on the 2011 UK census is available at `https://data.london.gov.uk/dataset/land-area-and-population-density-ward-and-borough/resource/77e9257d-ad9d-47aa-aeed-59a00741f301`. Last accessed Nov 6, 2016.

[3] Tobler, W. R. (1979). Smooth pycnophylactic interpolation for geographical regions. *Journal of the American Statistical Association*, **74** (367), 519–30. `doi:10.1080/01621459.1979.10481647`

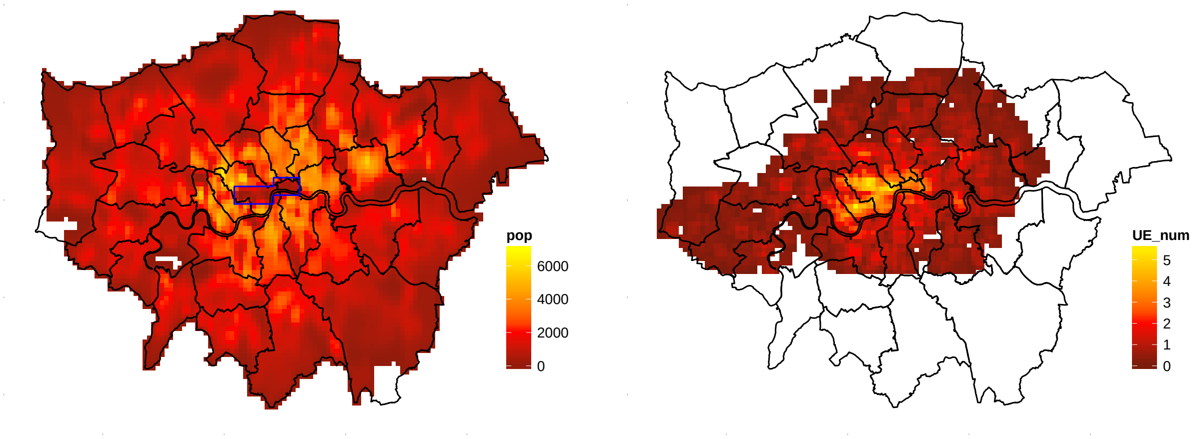

Figure S17: Comparison of population distribution (left) with the average scaled number of unique users present at midnight (right). The user number measures were averaged over the ten month data collection period. A most notable difference is present in the city center (area indicated by blue outline), where a high number of estimated people are present despite low population numbers. This can be explained by the prominent nightlife in this area and the presumption that people staying home are probably less active or already asleep; also note that smartphones which connect to the users' home WiFi network stop generating data communication, they would only be registered if the user places or receives a call or a text message.

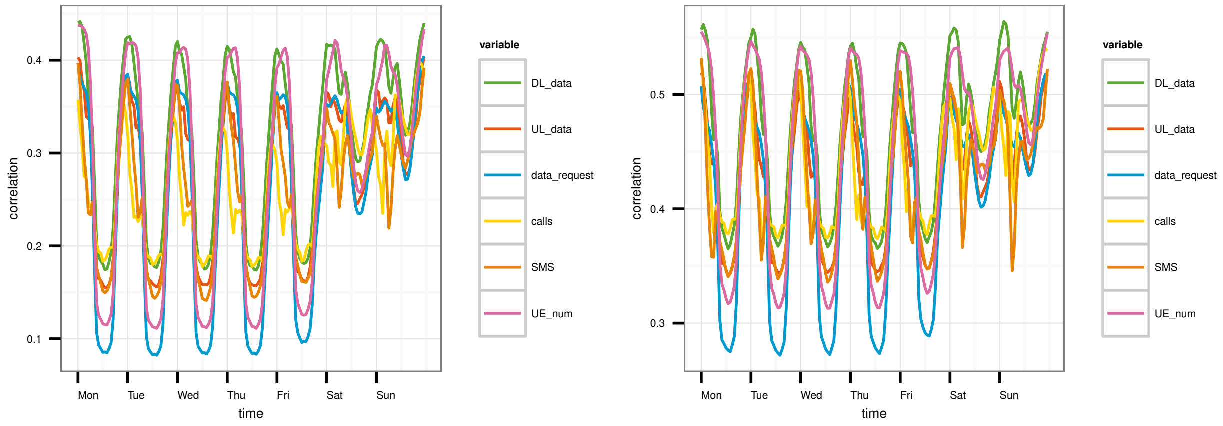

Figure S18: Spatial correlation among the typical week time series and the population distribution using the smoothed 500m by 500m grid. Pearson correlation coefficients were calculated using one the spatial distribution of activities in one hour intervals and the interpolated population distributions. It is notable that correlation is highest late night when considering data download volume and the number unique users registered for each antenna. The left panel displays measures calculated over the whole area, while the right panel displays correlation values excluding the city center which shows especially high activity even during night.
